# Gongying-Jiedu-Xiji recipe promotes the healing of venous ulcers by inhibiting ferroptosis via the CoQ-FSP1 axis

**DOI:** 10.3389/fphar.2023.1291099

**Published:** 2023-12-15

**Authors:** Yongpan Lu, Dejie Zhao, Ming Liu, Guoqi Cao, Chunyan Liu, Siyuan Yin, Ru Song, Jiaxu Ma, Rui Sun, Zhenjie Wu, Jian Liu, Yibing Wang

**Affiliations:** ^1^ First Clinical Medical College, Shandong University of Traditional Chinese Medicine, Jinan, China; ^2^ Jinan Clinical Research Center for Tissue Engineering Skin Regeneration and Wound Repair, Shandong Provincial Qianfoshan Hospital, The First Affiliated Hospital of Shandong First Medical University, Jinan, China; ^3^ Department of Vascular Surgery, Affiliated Hospital of Shandong University of Traditional Chinese Medicine, Jinan, China; ^4^ Department of Plastic Surgery, Shandong Provincial Qianfoshan Hospital, Shandong University, Jinan, China; ^5^ Department of Plastic Surgery, Shandong Provincial Qianfoshan Hospital, The First Affiliated Hospital of Shandong First Medical University, Jinan, China

**Keywords:** DDL, VU, ferroptosis, FSP1, CoQ, GPX4, lipid peroxidation

## Abstract

**Objective:** Gongying-Jiedu-Xiji recipe (DDL, batch number Z01080175) reduces body temperature, detoxifies, activates the blood circulation, reduces swelling, and dispels decay and pus. The aim of this study was to investigate the mechanism of action by which DDL functions in the treatment of venous ulcers (VUs).

**Methods:** Normal tissues as well as VU tissues before and after DDL treatment were collected from nine VU patients in the hospital with ethical approval. These three tissues were subjected to Prussian blue iron staining, immunoblotting, immunohistochemistry, immunofluorescence, and quantitative real-time PCR to detect the expression of ferroptosis suppressor protein 1 (FSP1), coenzyme Q (CoQ), 4-hydroxynonenal (4-HNE), and glutathione peroxidase 4 (GPX4). After successful validation of the heme-induced human foreskin fibroblast (HFF) ferroptosis model, lyophilized DDL powder was added to the cells, and the cells were subjected to viability assays, immunoblotting, flow cytometry, glutathione (GSH) and malonaldehyde (MDA) assays, electron microscopy and qPCR assays.

**Results:** Ferroptosis in VU tissues was stronger than that in normal tissues, and ferroptosis in VU tissues after DDL treatment was weaker than that before treatment. Inhibition of CoQ and FSP1 and transfection of FSP1 influenced the effects of DDL.

**Conclusion:** Our results suggest that DDL may promote healing by attenuating ferroptosis in VUs and that DDL may promote VU healing by modulating the CoQ-FSP1 axis.

## Highlights

• Firstly ferroptosis was found in venous diseases, venous ulcer tissues have strong ferroptosis, higher ferroptosis level than normal tissues.
• DDL can inhibit ferroptosis, and ferroptosis levels decreased after Dandelion detoxification lotion treatment of venous ulcers.
• Ferroptosis is very likely to be the main cause of recurrent venous ulcers, and may be a new signaling pathway for the treatment of venous ulcers in the future.
• DDL promotes venous ulcer healing by inhibiting ferroptosis through the CoQ-FSP1 axis, providing a new target for venous ulcer treatment.

## 1 Introduction

Patients often present with a history of chronic venous insufficiency (CVI), varicose veins in the lower extremities, and deep vein thrombosis in the lower extremities. Venous ulcers (VUs) are the final pathological outcome of CVI. According to data, the prevalence of VUs in our population is approximately 0.4%–1.3% (Arceo et al., 2002), the disease occurs in the foot and boot area, and patients often have a history of CVI or deep vein thrombosis in the lower extremities (Abelyan et al., 2018), which manifests as sunken and weak lower legs, dermatitis and eczema, hyperpigmentation, hardening of the skin (BG, 2001), and in the long term, the formation of intractable ulcers; in serious cases, patients may undergo amputation, or the risk of “cancer” is life-threatening (Alavi et al., 2016). The disease is difficult and costly to treat, and it is estimated that the United States spends up to $1 billion on VU treatment each year (Simka and Majewski, 2003). VUs have been a popular and difficult research topic in the field of trauma repair.

In recent years, a deeper understanding of the etiology and pathogenesis of VUs has been achieved, and their main etiology is hypertension of the venous system of the lower extremities (Chi and Raffetto, 2015); the causes of this condition mainly include venous reflux and/or venous reflux disorders in the lower extremities, penetrating venous insufficiency, and calf muscle pump insufficiency (Nüllen and Noppeney, 2010; O'Donnell, 2010; Recek, 2010). The “fibrin cuff” theory (Bonkemeyer Millan et al., 2019) and the “white blood cell trap” theory (Crawford et al., 2017) are also popular, but in recent years, studies have shown that the etiology of VUs seems to be related to the chronic inflammatory response to injury that is caused by persistent venous hypertension, and iron-containing erythropoiesis (Zhao and Chen, 2017), macrophage overexpression, and myofibroblast proliferation are closely associated with the development of ulcers (Krysa et al., 2012). However, the pathogenesis is complex, and no consensus has been reached. The development of CVI is associated with increased venous pressure due to inadequate venous return dynamics and venous structural disorders, and venous hypertension leads to extravasation of iron-containing hemoglobin (Raffetto and Mannello, 2014), which initiates a series of microscopic inflammatory and oxidative stress response mechanisms that result in VU development (Zheng and Conrad, 2020). Among these, ferroptosis due to oxidative stress injury that is associated with iron deposition is an area related to these microscopic mechanisms that remains to be understood (Stockwell et al., 2017).

Many natural products with therapeutic potential have been applied in clinical practice and have attracted increasing interest in recent years (Zhang and Wei, 2020). Moreover, an increasing number of natural products combined with other dressings and agents have achieved satisfactory results in the treatment of chronic wounds (Shedoeva et al., 2019; Hoff et al., 2021). In recent years, the exploration and research of the role of traditional Chinese medicine (TCM) in the prevention and treatment of trauma repair have attracted substantial attention. The Gongying-Jiedu-Xiji (DDL) recipe has been proven to be effective, safe and reliable by more than 40 years of clinical experience. Zhao Bo et al. (Zhao and Chen, 2017) applied DDL to treat VUs, and they achieved a cure rate of 28.95%, a significant healing rate of 71.05%, and an effective rate of 97.37%; thus, DDL has significant clinical effects. Studies on the use of DDL to treat lower extremity VUs have been limited to its use to improve antibacterial, anti-inflammatory and microcirculation effects. We studied the effects of DDL on matrix metalloproteinase 3 (MMP3) and matrix metalloproteinase inhibitor 1 (TIMP1) in the early stage. However, the use of DDL to ameliorate ferroptosis to promote VU healing has not been studied (Zhao and Xu, 2020).

Currently, the pharmacological treatment of VUs is gradually increasing, and scholars have found that human recombinant epidermal growth factor (hrEGF) can promote faster healing of VUs by stimulating granulation tissue and accelerating the rate of epithelialization (Cacua Sanchez et al., 2023). Oleuropein is an active metabolite extracted from olive leaves that has antioxidant effects and can reduce tissue damage. Research has shown that oleuropein can effectively shorten the healing time of VUs with the help of nanomaterials attached to oleuropein, but the poor pharmacokinetics of oleuropein have limited its use in clinical applications (Alquraishi et al., 2023). DDL has not yet been found to have deficiencies in terms of pharmacokinetics. It has been found that pentoxifylline can promote VU healing, and the main mechanisms are a reduction in the erythrocyte sedimentation rate, a reduction in plasma viscosity, a reduction in leukocyte adhesion on the venous vessel wall, promotion of collagen production, and blockade of the effect of tumor necrosis factor-α on fibroblasts (Chen et al., 2023). However, pentoxifylline often causes gastrointestinal adverse reactions (Jull et al., 2002). DDL has not yet resulted in adverse reactions, and its clinical application is safer. One previous study revealed that after treatment of VUs with an aqueous solution of Phellodendron Bark, the level of NF-κB decreased, while the levels of IκBα and TNFAIP3/A20 increased (WANG Xi and Zhao, 2017). Other mechanisms of drugs used to treat VUs involve VEGF, transforming growth factor (CDmTGF-β1), interleukin-1β (IL-1β) and other molecules (Raffetto et al., 2016).

In this study, we collected normal tissues and VU samples for experiments to verify the differential expression of ferroptosis-related genes and collected VU tissue samples before and after DDL treatment to experimentally verify these findings. Then, hemin was used to establish a VU cell model to perform cellular experiments, and the mechanism of action of DDL was investigated *in vitro*. The main purpose of this study was to investigate the mechanism of action of DDL in treating VU and to provide an experimental basis for its clinical application.

## 2 Materials and methods

### 2.1 Reagents

DDL is currently used as an agreed-upon formula in Shandong Provincial Hospital of Traditional Chinese Medicine (Lu medicine production batch number Z01080175). A protease inhibitor cocktail (Cat No. HY-K0010) was purchased from MedChemExpress (NJ, USA). Enhanced BCA protein assay kits (P0010S) were ordered from Beyotime Institute of Biotechnology (Shanghai, China). A SteadyPure Quick RNA Extraction Kit (Code No. AG21023) was purchased from Accurate Biotechnology Co., Ltd. (Hunan, China). The relative concentration of the solvent dimethyl sulfoxide (DMSO; CAS. 67–68-5; MP Biomedicals, LLC, USA). 4-Chlorobenzoic acid (4-CBA) (No. 135585) was purchased from Sigma‒Aldrich (Shanghai, China). Inhibitor-Ferroptosis suppressor protein 1 (iFSP1) (Cat. No: HY-136057) was purchased from MedChemExpress. Ferrostatin-1 (Cat. No.: HY-100579) was purchased from MedChemExpress.

### 2.2 Preparation of DDL and high-performance liquid chromatography (HPLC) analysis

The chemical reagents used in this experiment were all high-performance liquid chromatography (HPLC) analytical grade. DDL lyophilized powder was prepared according to classical reports (Du et al., 2021). The specific contents and properties of the ten botanical drugs are listed in Table 1, and the full species name, authorities, family and drug name are included (Rivera et al., 2014). DDL (Production Batch No. Z01080175) was packaged, produced and supplied by the Preparation Room of the Affiliated Hospital of Shandong University of Traditional Chinese Medicine. A technical report was provided by the Preparation Office of the Affiliated Hospital of Shandong University of Traditional Chinese Medicine, with Taraxacum mongolicum Hand.-Mazz as the main botanical drug, followed by Phellodendron chinense C.K. Schneid, Sophora flavescens Aiton, Forsythia suspensa (Thunb.) Vahl and Momordica cochinchinensis (Lour.) Spreng, followed by *Lonicera japonica* Thunb, Paeonia × suffruticosa Andrews, Angelica dahurica (Hoffm.) Benth. & Hook.f. ex Franch. & Sav, Paeonia lactiflora Pall and Glycyrrhiza uralensis Fisch. ex DC. The three parts were composed at a ratio of 7.5:3:2.5 weight (w/w). These ten botanical drugs were identified by Prof. Hongsong Qin, and the voucher samples were kept in the Department of Pharmacy, Affiliated Hospital of Shandong University of Traditional Chinese Medicine.

**TABLE 1:** Characteristics of the ten botanical drugs in the DDL pharmaceutical product.

Chinese name	Botanical name	Genus family	Batch number	Medicinal parts	Origin	Weight (g)
Pugongying	TaraxacummongolicumHand.-Mazz	Asteraceae	220302	Dried herb	Shandong, China	30
Huangbo	Phellodendron chinense C.K.Schneid	Rutaceae	2204030282	Dried bark	Chongqing, China	12
Kushen	Sophora flavescens Aiton	Fabaceae	2203120051	Dried root	Jilin, China	12
Lianqiao	Forsythia suspensa (Thunb.) Vahl	Oleaceae	2203150072	Dried fruit	Shanxi, China	12
Mubiezi	Momordica cochinensis (Lour.)Spreng	Cucurbitaceae	2202270402	Dried ripe seed	Guangxi, China	12
Jinyinhua	Lonicera japonica Thunb	Caprifoliaceae	2203180032	Dried stem and branch	Shandong, China	10
Mudanpi	Paeonia× suffruticosa Andrews	Paeoniaceae	2203150152	Dried root bark	Anhui, China	10
Baizhi	Angelica dahurica (Fisch.ex Hoffm.)Benth.et Hook.f	Apiaceae	220202	Dried root	Sichuang, China	10
Chishao	Paeonia lactiflora Pall	Paeoniaceae	2203050012	Dried root	Neimenggu, China	10
Gancao	Glycyrrhiza uralensis Fisch. ex DC	Fabaceae	2203020092	Dried root and rhizome	Gansu, China	10

The plant name was verified using http://mpns.kew.org/mpns-portal/ and http://www.plantsoftheworldonline.org

Preparation room processing method: DDL preparation followed the guidelines of prescription science outlined in the Ministry of Education’s general higher education “13th Five-Year Plan” national planning materials, using the traditional method of boiling botanical drugs, placing drugs in a decoction machine to decoct the dried botanicals, and immersing the above ten botanical drugs in pure water at 20 times the volume of the botanicals (v/w) of pure water for 30 min, heated to boiling and kept for 30 min, filtered and collected. The decoction was boiled again as described above, and the liquid was collected, mixed with the initial liquid and concentrated to 250 mL/bag for packaging. DDL were first filtered 4 times, and the filtrates were obtained and prefrozen using a vacuum freeze dryer (LGJ-18A type freeze dryer, No. 030352) at −42.5°C for 4 h. Then, sublimation drying was carried out, and low-temperature sublimation drying at −20°C was performed for 14 h. Finally, resolution drying was carried out at 30°C for 14 h. The filtered extracts were concentrated and further freeze-dried into fine powder with a final yield of 18.3%. The extraction rate was 9.8%, i.e., 9.8 g per 100 g of drug, as determined by acid dye colorimetry (Yin et al., 2022).

In order to identify the major metabolites of DDL, ultra performance liquid chromatography coupled with high resolution mass spectrometry (UPLC-Q-TOF/MS) was utilized for mass spectrometry data acquisition of DDL. Weigh 0.5 g of sample in a 50 mL stoppered conical flask, add 10% methanol 10 mL, shake well, ultrasonic (power 300 W, frequency 40 kHz) for 30 min, high speed centrifugation (12000 rpm) for 5 min, the supernatant was extracted. W, frequency 40 kHz) for 30 min, centrifuged at high speed (12000 rpm) for 5 min, and the supernatant was extracted. A Waters H-Class ultra-high performance liquid chromatograph (Waters Technology Co., Ltd.) was used. The chromatographic separation was performed on an Agilent Poroshell 120 AQ-C18 column (2.1 × 100 mm, 2.7 μm) at 30°C with a flow rate of 0.5 mL/min and an injection volume of 2 μL at detection wavelengths of 254 nm and 190–400 nm. The separation was carried out using a linear gradient mobile phase as the mobile phase. The ratio of mobile phases was acetonitrile in phase A and 0.1% formic acid aqueous solution in phase B. The mobile phase gradients were (0–30 min, 3%–16% A, 97%–84% B; 30–45 min, 6%–40% A, 84%–60% B; 45–50 min, 40%–90% A, 60%–10% B; 50–53 min, 90% A, 10% B; 53–53.1 min, 90%–3% A, 10%–97% B; 53.1–56 min 3%A, 97%B) (Heinrich et al., 2022).

### 2.3 Cell culture, processing, and transfection

Normal human foreskin fibroblasts (HFFs) were purchased from Cell Research (Shanghai, China) and passaged in high-glucose Dulbecco’s modified Eagle’s medium (DMEM; Gibco, Thermo Fisher Scientific, MA, USA) supplemented with 15% FBS. Human umbilical vein endothelial cells (HUVECs) were purchased from MeisenCTCC (Zhejiang, China) and passaged in high-glucose DMEM supplemented with 10% FBS. Both cell types were cultured at 37°C with 5% CO_2_. HFFs and HUVECs were cultured in medium (Cellntec, Switzerland) supplemented with different concentrations of hemin (Sigma, Germany; 10 μmol/L, 20 μmol/L, 30 μmol/L, 40 μmol/L, and 50 μmol/L) for 48 h. HFFs and HUVECs were cultured in medium (Cellntec, Switzerland) supplemented with different concentrations of DDLLP (10 μmol/L, 20 μmol/L, 50 μmol/L, 100 μmol/L, and 200 μmol/L) for 48 h. For FSP1 overexpression experiments, HFFs were infected with the FSP1 plasmid and Empty vector plasmid (Shandong Gene&Bio Co., Ltd., China) for 36 h. The cells were harvested for RNA and protein extraction and assay, and cells were grown on slides for IF validation.

The sequences that were used are shown below.

FSP1-F 5′-CGC​AAA​TGG​GCG​GTA​GGC​GTG-3′

FSP1-R 5′-TAG​AAG​GCA​CAG​TCG​AGG -3′

### 2.4 Cell viability assay

We determined cell viability as previously described at 1–48 h after hemin exposure. DiYO™-3 (AAT Bioquest; 17581, 1/1000) was added to the cells that were exposed to different treatments. HFFs exposed to different treatments were seeded in a 96-well plate (Corning Incorporated, USA) at an initial density of 3000 cells per well, and then the plate was placed in an IncuCyte S3 Live-Cell Analysis System, where real-time images were captured every 1 h for 48 h. Photographs of the cells were taken in three separate regions of each well using a ×10 objective. The values from the three regions of each well were pooled and averaged across three replicates. Additionally, using the IncuCyte S3 program (Essen BioScience, Ann Arbor, MI, USA), cells that were labeled with DiYOTM-3 were counted. Cell survival was measured and normalized to that at 0 h.

### 2.5 Assessment of drug toxicity and cell proliferation

Cells were seeded in 96-well plates at 4 ×10^5^ cells per well, cultured in medium with one of several concentrations of DDLLP (0 μg/mL, 1 μg/mL, 10 μg/mL, 100 μg/mL, 200 μg/mL, or 1 mg/mL) and observed. Multiple 96-well plates were used for CCK-8 analyses (Dojindo, Japan, 1/10), and some were placed in an IncuCyte S3 Live-Cell Analysis System for testing. A CCK-8 assay was used to assess the viability of treated cells. After culturing for 48 h, the medium was removed, 100 μL of fresh medium and 10 μL of CCK-8 were added to each well, and cell viability was measured after incubation at 37°C for 1 h. A Spark microplate reader (Tecan, Austria) was used to measure cell viability at an absorbance of 450 nm. IncuCyte S3 Live-Cell Analysis System analysis was performed as previously described.

### 2.6 Patient and tissue samples

Tissue samples were proactively collected from nine patients with VUs who were admitted to the Shandong Provincial Hospital of Traditional Chinese Medicine in Jinan, China. Normal tissue, pre-DDL-treated VU tissue, and post-DDL-treated VU tissue were collected from each of the nine patients. To perform molecular analyses, half of these tissues were divided into two pieces and immediately frozen in liquid nitrogen in 1.5 mL snap-cap tubes (Yin et al., 2022). This investigation followed the principles outlined in the Declaration of Helsinki. All experiments were carried out in accordance with the rules and regulations and were approved by the ethics committees of the Affiliated Hospitals of Shandong University of Traditional Chinese Medicine and Shandong Provincial Hospital of Traditional Chinese Medicine (approval number: AF/SC-08/02.0). The other half of the tissues were used for immunohistochemistry as described.

### 2.7 Protein extraction

Proteins were extracted from VU tissue samples that were stored at −80°C using the Minute Total Skin Tissue Protein Extraction Kit. After cutting the tissues with scissors, the samples were placed in centrifuge tube columns. Then, 100 mg of crushed protein powder was added to each tissue sample. Each skin tissue sample was mixed with 200 µL of lysis solution containing a protease inhibitor (1:100). A grinding rod was used to homogenize the tissue for 5 min, and then 100 µL of lysate was mixed with a protease inhibitor and homogenized for an additional 2 min. The homogenized samples were placed in a benchtop centrifuge with a centrifuge column and centrifuged at 12000 rpm for 1 min. Then, the column was discarded. The supernatants in the casing were collected and used as whole protein extracts. These supernatants were transferred to fresh centrifuge tubes and stored at −80°C for future experiments; the protein levels were measured using the BCA Protein Assay Kit. The process for cellular protein extraction was similar, but it differed from the process for tissue protein extraction. To measure relative protein expression levels, treated HFFs were fully lysed in RIPA buffer (Thermo Fisher Scientific) to obtain protein lysates. Protease inhibitors and phosphatase inhibitors (1:100; MedChemExpress) were added during protein extraction, and a Pierce BCA Protein Analysis Kit (Thermo Fisher Scientific) was used to measure protein concentrations.

### 2.8 Western blotting analysis

Western blotting analysis was performed to measure protein expression levels. SDS‒PAGE was used to separate protein samples, which were then transferred to PVDF membranes. The membranes were blocked in 5% skim milk and treated overnight at 4°C with the appropriate primary antibodies. The samples were incubated for 1 h at room temperature with horseradish peroxidase-conjugated secondary antibodies (1:5000 dilution; Cell Signaling Technology), proteins were detected, and their expression levels were analyzed using an iBright FL1500 imaging system (Invitrogen) and Super Signal West Femto Maximum Sensitivity Substrate (Thermo Fisher Scientific, Invitrogen). Densitometry analysis was performed using ImageJ software. The protein expression levels were normalized to those of the endogenous control tubulin or GAPDH. Information about the anti-FSP1 (Thermo Fisher Scientific; PA5-88365), anti-GPX4 (Abcam; ab125066), anti-CoQ (Santa Cruz Biotechnology, Inc. ; sc-517107), anti-ACSL4 (Abcam; ab155282), anti-Tubulin (Proteintech; 66031-1-Ig), and anti-GAPDH (Cell Signaling Technology; WB: 1/1000) antibodies are provided.

### 2.9 Immunohistochemical (IHC) staining

The remaining portions of the tissue samples were fixed in 10% paraformaldehyde, embedded in paraffin, and sectioned at a thickness of 3 µm for histochemical studies that included hematoxylin-eosin (H&E) and IHC staining. Paraffin sections were deparaffinized, rehydrated, subjected to antigen retrieval, subjected to endogenous peroxidase inhibition, and blocked with goat serum (#SP-9001; ZSGB-BIO, Beijing, China). The skin tissue sections were then incubated at 4°C overnight with primary antibodies against FSP1 (Thermo Fisher Scientific; PA5-88365), GPX4 (Servicebio; GB114327), and CoQ (Abcam; ab220914) at a dilution of 1:200. On the second day, all the sections were treated with biotin-labeled goat anti-mouse IgG polymer for 15 min at room temperature and with horseradish enzyme-labeled streptavidin working solution for 15 min. Finally, the sections were stained with diaminobenzidine (DAB; #ZLI-9018, ZSGB-BIO) and hematoxylin (CAS 517-28-2; Beijing Solarbio Science & Technology). After deparaffinization and rehydration, the sections were stained with hematoxylin and eosin (CAS. 17372-87-1; Beijing Solarbio Science & Technology) following the manufacturer’s instructions. Afterward, all of the sections were dehydrated, cleaned, and sealed. An Olympus IX73 microscope (Olympus, Tokyo, Japan) was used to observe and capture the images. The rate of positive staining was calculated using ImageJ software.

### 2.10 Perls staining and diaminobenzidine (DAB) staining

Paraffin sections were deparaffinized and rehydrated; then, the slides were incubated with xylene I for 20 min, xylene II for 20 min, absolute ethanol I for 5 min, absolute ethanol II for 5 min, and 75% alcohol for 5 min, followed by washing with tap water and distilled water 3 times. For Prussian blue staining, 2% potassium ferrohydride was mixed with 2% hydrochloric acid in equal proportions, and the slides were incubated with the mixed solution for 30 min and then washed twice with distilled water. The samples were stained with DAB color droplets for approximately 5–10 min, and the degree of color development was controlled under a microscope. After removing the staining solution, the samples were rinsed once with 0.01 mol/L PBS solution and washed with distilled water three times. The samples were stained with hematoxylin dye for 1 min, washed with tap water, differentiated with hydrochloric acid aqueous minutes, washed with tap water, immersed in an aqueous ammonia solution, and then washed with tap water. The slides were incubated with absolute ethanol I for 5 min, absolute ethanol II for 5 min, absolute ethanol III for 5 min, xylene Ⅰ for 5 min, and xylene Ⅱ for 5 min to render the slides transparent and then sealed with neutral balsam. An Olympus IX73 microscope (Olympus, Tokyo, Japan) was used to observe and capture the images. The rate of positive staining was calculated using ImageJ software.

### 2.11 Immunofluorescence (IF) staining

To dewax the paraffin sections with water, the sections were incubated with environmentally friendly dewaxing solution I for 10 min, environmentally friendly dewaxing solution II for 10 min, environmentally friendly dewaxing solution III for 10 min, anhydrous ethanol I for 5 min, anhydrous ethanol II for 5 min, and anhydrous ethanol III for 5 min. Then, the sections were washed with distilled water. For antigen repair, excessive buffer evaporation was avoided, and the film was not allowed to dry. After antigen repair was complete, the samples were allowed to cool naturally. The slides were placed on a decolorization shaker in PBS (pH 7.4) and washed with shaking 3 times for 5 min each. After lightly shaking a section, a circle was drawn around the tissue with a histochemical pen, and BSA was applied dropwise and incubated for 30 min.

Mixed reagents of the first and second primary antibodies were added as follows: two different sources of primary antibodies were mixed and configured, matched primary antibodies were added dropwise, and the sections were incubated flat in a wet box at 4°C overnight. Then, the sections were placed in PBS (pH 7.4) and washed with shaking 3 times for 5 min each on a decolorization shaker. Then, the sections were incubated for 50 min at room temperature in the dark with the corresponding secondary antibodies. Nuclei were stained with DAPI (#62248; Thermo Fisher Scientific). The slides were placed in PBS (pH 7.4) and washed three times with shaking for 5 min each. The slides were then incubated for 10 min at room temperature in the dark with DAPI staining solution. Tissue autofluorescence was quenched by incubating the slides in PBS (pH 7.4) and washing three times for 5 min each on a decolorized shaker. After 5 min, autofluorescence quencher B solution was added, and the slides were rinsed with running water for 10 min. The slides were sealed with an anti-fluorescence-quenching sealant. We carried out an IF assay on cells once more. The cells were fixed in 4% paraformaldehyde for 15 min at room temperature, washed 3 times in PBS, permeabilized in 0.5% Triton X-100 and PBS for 10 min, and then blocked with BlockAid Blocking Solution (Invitrogen) for 1 h. The cells were incubated with primary antibodies overnight at 4°C and then with an anti-mouse Alexa Fluor-488 (diluted 1/200, Cell Signaling Technology) secondary antibody at room temperature for 1 h. Finally, nuclei were stained with Hoechst (1:10,000; Invitrogen, Thermo Fisher Scientific) at room temperature for 30 min. Finally, a ZEISS Cell Discoverer7 with LSM900 and Airyscan2 (Carl Zeiss Microscope GmbH, Jena, Germany) was used to view the slides and record the findings. The primary antibodies that were utilized were anti-FSP1 (Santa Cruz Biotechnology, Inc. ; sc-377120), anti-4-HNE (Abcam; ab48506), anti-GPX4 (Servicebio; GB114327), and anti-CoQ (Abcam; ab220914) antibodies. The secondary antibodies that were used included anti-mouse Alexa Fluor-488 (Servicebio; GB25301), anti-rabbit Alexa Fluor-488 (Servicebio; GB25303), anti-rabbit Alexa Fluor-594 (Jackson; 111-585-003), and anti-mouse Alexa Fluor-594 (Jackson; 115-585-003).

### 2.12 Observation of mitochondrial morphology by transmission electron microscopy (TEM)

Cells were grouped according to the different treatments. Cell pellets were collected by centrifugation. TEM fixative (Servicebio; G1102) was added to the tubes, and the pellets were resuspended in the fixative. After preembedding in agarose, the fixed cells were centrifuged. Then, 0.1 M PB (pH 7.4) was added to the tubes after the supernatants were discarded, and the pellets were resuspended and washed in PB for 3 min. This washing step was repeated 3 times. The 1% agarose solution was prepared by heating and dissolving in advance. After being cooled, the agarose solution was added to an EP tube. Before the agarose polymerized, the pellet was suspended with forceps and wrapped in the agarose. Agarose blocks containing the samples were postfixed with 1% OsO4 in 0.1 MPB (pH 7.4) for 2 h at room temperature in the dark. After removing OsO4, the tissues were rinsed with 0.1 M PB (pH 7.4) 3 times for 15 min each. The samples were dehydrated at room temperature as follows: incubation with 30% ethanol for 20 min, 50% ethanol for 20 min, 70% ethanol for 20 min, 80% ethanol for 20 min, and 95% ethanol for 20 min, followed by two washes with 100% ethanol for 20 min each and two washes with acetone for 15 min each. Resin penetration and embedding were performed as follows: the samples were incubated with acetone:EMbed 812 at a 1:1 ratio for 2–4 h at 37°C, incubated with acetone:EMBed 812 at a 1:2 ratio overnight at 37°C, and incubated with pure EMBed 812 for 5–8 h at 37°C. Pure EMBed 812 was poured into the embedding models, and the tissues were immersed in pure EMBed 812 and incubated in a 37°C oven overnight. The embedding models containing the resin and samples were placed in a 65°C oven and allowed to polymerize for more than 48 h. Then, the resin blocks were removed from the embedding models and incubated at room temperature. The resin blocks were cut into 60–80 nm sections on an ultramicrotome, and the tissues were removed and placed on 150 mesh cuprum grids with Formvar film. A 2% uranium acetate saturated alcohol solution was used to stain the samples for 8 min in the dark, and the samples were washed with 70% ethanol 3 times and then washed with ultrapure water 3 times. Then, 2.6% lead citrate was used to avoid CO_2_ staining for 8 min, and the sections were rinsed with ultrapure water 3 times. After drying with filter paper, the cuprum grids were placed into the grid board and dried overnight at room temperature. The cuprum grids were observed by TEM (Hitachi, HT7800), and images were captured.

### 2.13 MDA level evaluation

MDA levels were calculated following the instructions of the Lipid Peroxidation MDA Assay Kit (AAT Bioquest; 15991). Test samples (50 µL) or MDA standards were prepared and added. MDA Blue™ (10 µL) was prepared and added. Then, the samples and standards were incubated for 10–30 min at room temperature. Then, 40 mL of the reaction solution was added, and the OD was measured at 695 nm.

### 2.14 GSH level evaluation

GSH levels were quantified according to the instructions of the reduced glutathione (GSH) assay kit (Servicebio; G4305). The cells were carefully removed with a cell scraper and centrifuged for 10 min at 3000 rpm with the appropriate amount of culture medium. The cells were resuspended using a protein removal reagent (approximately 100–200 μL of reagent per 1 × 10^7 cells). The cells were lysed by sonication or on ice; after treatment, the supernatants were removed by centrifugation at 10000 *g* for 10–15 min at 4°C. The standards were then prepared by mixing the protein removal reagent and ultrapure water at a ratio of 1:9, and the reduced glutathione standard (1 mmol/L) was diluted for use. Then, the samples to be tested were compared to the diluted standards, and the data were analyzed using the standard conversion procedure.

### 2.15 Flow cytometry analysis of intracellular lipid peroxidation and divalent iron ion levels

Multiple groups of cells were treated with different treatments, digested with trypsin (Gibco, Canada), collected into 2 mL sample tubes, and centrifuged (1500 × g and 5 min). BODIPY 581/591 C11 (Invitrogen, USA, 1:1000) and FerroOrange (Dojindo, Japan, 1:500) probes were used to determine intracellular lipid peroxidation levels and divalent iron ion levels, respectively, in cells. The cells were first stained with the BODIPY 581/591 C11 or FerroOrange probe and then with eBioscience Flow Cytometry Staining Buffer (the pellet was resuspended in 300 µL of the buffer after centrifugation) at 37°C for 40 min, and then, the cells were analyzed with a CytoFLEX flow cytometer (Beckman Coulter, Indianapolis, CA). First, we distinguished dead cells, cell debris, and live cells by adjusting the voltage so that the dead cells and cell debris would accumulate in the lower left position of the FSC-SSC voltage gate. Then, the live cells were examined, and values within the FITC gate and PE gate were determined. Then, the cells were treated with the lipid peroxidation probes C11-BODIPY and FerroOrange and examined; the values in the FITC gate and PE gate were determined. Oxidation of the polyunsaturated butadiene fraction of C11-BODIPY resulted in a shift in the fluorescence emission peak from ∼ 590 nm to ∼ 510 nm, as detected in the PE and FITC channels. FerroOrange was excited at 543 nm, and its emission (580 nm) was detected in the PE channel. The relative levels of lipid peroxidation and divalent ferric ions were quantified by calculating the arithmetic mean of the FITC/PE and PE values, respectively, across three experiments.

### 2.16 RNA isolation and quantitative real-time PCR (qRT‒PCR)

Total RNA was extracted using RNAiso Plus (9109, Takara, Japan) according to the manufacturer’s protocol. Sketch™ RT Master Mix (RR036A, Takara, Japan) was used to synthesize complementary DNA via the reverse transcription of RNA into DNA. Quantitative real-time PCR (qRT–PCR) experiments were performed using TB-Green™ Premix™ II (RR820A, Takara, Japan). GAPDH was used as an endogenous reference gene. Relative gene expression was determined using the 2^−ΔΔCT^ method. The following is a list of the PCR primer sequences that were used to analyze the skin tissues.

GAPDH-F 5′-GGA​AGC​TTG​TCA​TCA​ATG​GAA​ATC-3′

GAPDH-R 5′-TGA​TGA​CCC​TTT​TGG​CTC​CC-3′

FSP1-F 5′- CTC​CGT​GGA​GAC​AGG​GTT​CG -3′

FSP1-R 5′- GGT​TCT​TCA​GGT​CTA​TCC​CCA​CTA -3′

CoQ-F 5′-CTC​ATG​CGG​TTG​GAC​AAG​C-3′

CoQ-R 5′-CCT​GCT​CCA​CGC​ATC​AGA​ATA -3′

GPX4-F 5′-CCG​CTG​TGG​AAG​TGG​ATG​AAG -3′

GPX4-R 5′-CTT​GTC​GAT​GAG​GAA​CTT​GGT​GAA -3′

The following primer sequences were used to verify the establishment of overexpressing cells.

Actin-F 5′- TGG​CAC​CCA​GCA​CAA​TGA​A -3′

Actin-R 5′- CTA​AGT​CAT​AGT​CCG​CCT​AGA​AGC​A -3′

FSP1-F 5′- CAA​GAT​CAA​CAG​CTC​CGC​CTA​C -3′

FSP1-R 5′- AGG​TGC​TCG​TTC​ACT​CTC​AGA -3′

### 2.17 Inhibitors of the CoQ or FSP1 pathway

Cells were seeded in six-well plates, and after 24 h of culture, an inhibitor of CoQ (4-chlorobenzoic acid, Sigma‒Aldrich; 20 μM, 14 h) was added to the cells.

Cells were seeded in six-well plates, and after 24 h of cell culture, FSP1 (iFSP1, MedChemExpress; 20 μM, 12 h) was added to the cells.

### 2.18 Statistical analysis

The data for all the experiments are shown as the means ± standard deviations (SD) of three biological replicates, and all the data analyses were performed using GraphPad Prism version 9.0.0 (GraphPad Software, San Diego, CA). Statistical analysis to determine the significance of the differences between groups was performed using Student’s t-test. Asterisks indicate significant differences between conditions in each group. *p* values are indicated as *p < 0.05, **p < 0.01, ***p < 0.001.

## 3 Results

### 3.1 Mass spectrometry analysis of metabolites in DDL

**FIGURE 1:**
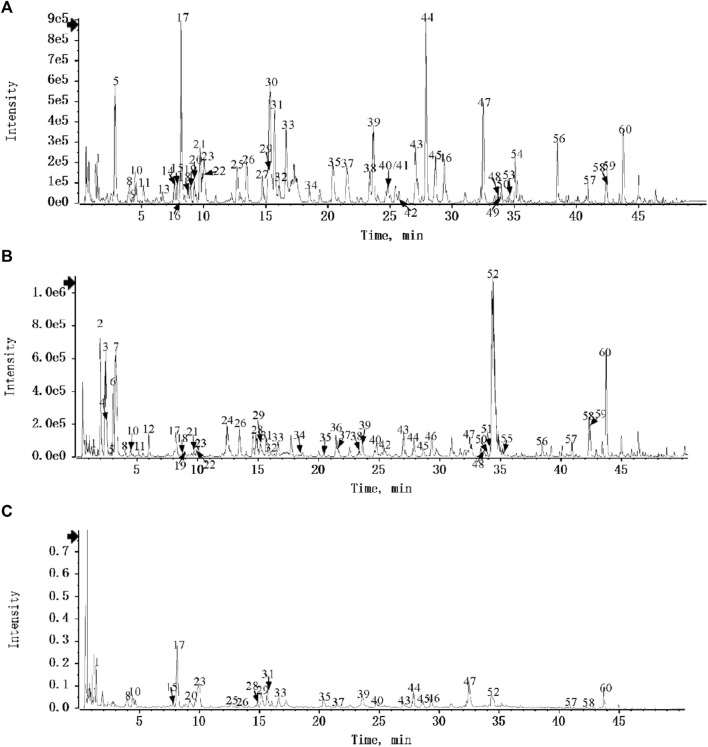
Identification of metabolites of DDL. **(A)** UPLC-HRMS Base Peak Ion Flow Chart (BPC) of DDL - Negative Ion Mode. **(B)** UPLC-HRMS Base Peak Ionogram (BPC) of DDL - Positive Ion Mode. **(C)** UPLC UV chromatogram of DDL - UV 254 nm.

DDL was analyzed by UPLC-Q-TOF/MS, and based on the multistage mass spectral information of the samples, combined with the database of natural product high resolution mass spectrometry (NP-HRMS) and the related literature, 60 metabolites were identified from DDL (Table 2). The data acquisition software was Analyst TF 1.7.1, and the data processing software was Peakview 1.2. We investigated the UPLC-HRMS basal peak ion mobility chromatograms (BPC)-negative ion mode (Figure 1A) in DDL, and the UPLC-HRMS basal peak ion mobility chromatograms (BPC)-positive ion mode (Figure 1B) in DDL. UPLC UV chromatogram in DDL - UV 254 nm (Figure 1C). The labeled number in Figure 1 is the Serial No. in Table 2. The mass spectrometry data were preferentially matched with the Natural Products HR-MS/MS Spectral Library 1.0 (NPHSL) database during the identification. The NPHSL database contains multistage mass spectra of standards from Shanghai Dagit Pharmaceutical Technology Co. or other sources, including different acquisition modes, different addition ions, different collision energies, etc. The information of the compounds is comprehensive, and all of them are the maps of the actual standards, without any simulated and speculative maps, so the matching results are highly accurate. Metabolites not included in the database were identified based on literature reports, mass spectrometry cleavage patterns, etc .,(Zhang et al., 2018). The results were summarized in Table 2. Based on the peak area values, the higher peak area values indicated the higher content of metabolites in the DDL, and the top 8 contents were Berberine, Forsythoside A, Paeoniflorin, Vogeloside, Secologanin, and Piscidic acid, in that order, Isochlorogenicacid C and Matrine.

**TABLE 2:** Metabolites were predicted of DDL in the NPHSL database.

Serial No.	Molecular formula	Molecular weight	Metabolite	MS/MS data	Attribution (Botanical name)	Peak area
52	C_20_H_18_NO_4_+	336.12	Berberine	336.1242; 320.0919; 292.0970; 278.0812	Phellodendron chinense C.K.Schneid	48615817
44	C_29_H_36_O_15_	624.21	Forsythoside A	623.2000; 461.1677; 443.1677; 161.0255; 133.0299	Forsythia suspensa (Thunb.) Vahl	43983231
30	C_23_H_28_O_11_	480.16	Paeoniflorin	449.1430; 327.1057; 165.0549; 121.0288	Paeonia× suffruticosa Andrews/Paeonia lactiflora Pall	42595046
33	C_17_H_24_O_10_	388.14	Vogeloside	433.1332; 387.1284; 225.0755; 155.0343	*Lonicera japonica* Thunb	27353834
31	C_17_H_24_O_10_	388.14	Secologanin	433.1340; 387.1289; 155.0348	*Lonicera japonica* Thunb	26778773
5	C_11_H_12_O_7_	256.06	Piscidic acid	255.0505; 193.0505; 179.0349; 165.0555; 147.0448	Sophora flavescens Aiton	24074238
47	C_25_H_24_O_12_	516.13	Isochlorogenicacid C	515.1173; 353.0857; 191.0550; 179.0339; 173.0446	TaraxacummongolicumHand.-Mazz/*Lonicera japonica* Thunb	23997874
2	C_15_H_24_N_2_O	248.19	Matrine	249.1960; 176.1067; 148.1119	Sophora flavescens Aiton	23921504
39	C_29_H_36_O_15_	624.21	Forsythoside I	623.1959; 461.1641; 283.0595; 179.0341; 161.0237	Forsythia suspensa (Thunb.) Vahl	20539664
23	C_16_H_22_O_10_	374.12	Secologanic acid	373.1132; 193.05041; 149.0606; 119.0348	*Lonicera japonica* Thunb	19678056
17	C_16_H_18_O_9_	354.10	Chlorogenic acid	353.0896; 19.0571; 173.0469; 135.0460	TaraxacummongolicumHand.-Mazz/*Lonicera japonica* Thunb	18464903
35	C_22_H_18_O_12_	474.08	Chicoric acid	311.0410; 293.0302; 219.0287; 179.0345; 149.0091; 135.0450	TaraxacummongolicumHand.-Mazz	16338813
37	C_21_H_22_O_9_	418.13	Liquiritin	417.1193; 255.0661; 135.0092; 119.0510	Glycyrrhiza uralensis Fisch. ex DC	16162642
21	C_16_H_22_O_11_	390.12	Secoxyloganic acid	389.1092; 345.1185; 209.0458; 183.0666; 165.0558; 121.0660	*Lonicera japonica* Thunb	15318324
45	C_25_H_24_O_12_	516.13	Isochlorogenicacid B	515.1189; 353.0862; 335.0755; 191.0552; 179.0343; 173.0449	TaraxacummongolicumHand.-Mazz/*Lonicera japonica* Thunb	14566760
7	C_15_H_24_N_2_O_2_	264.18	Oxymatrine	265.1913; 247.1807; 205.1337; 148.1120	Sophora flavescens Aiton	14436810
43	C_29_H_36_O_15_	624.21	Forsythoside H	623.2006; 461.1677; 179.0360; 161.0253	Forsythia suspensa (Thunb.) Vahl	14347314
46	C_25_H_24_O_12_	516.13	Isochlorogenicacid A	353.0880; 191.0565; 179.0353; 135.0456	TaraxacummongolicumHand.-Mazz/*Lonicera japonica* Thunb	13905589
60	C_42_H_62_O_16_	822.40	Glycyrrhizic acid	821.3935; 351.0547; 193.0337	Glycyrrhiza uralensis Fisch. ex DC	13104754
24	C_20_H_24_NO_4_+	342.17	Phellodendrine	342.1711; 192.1027; 177.0791; 149.0835	Phellodendron chinense C.K.Schneid	12267702
6	C_15_H_22_N_2_O_2_	262.17	Oxysophocarpine	263.1754; 245.1644; 203.1176; 150.1278	Sophora flavescens Aiton	12204888
3	C_15_H_22_N_2_O	246.17	Sophocarpine	247.1801; 179.1540; 150.1274; 136.1118	Sophora flavescens Aiton	11897949
26	C_23_H_28_O_11_	480.16	Albiflorin	327.1025; 165.0579; 121.0294	Paeonia× suffruticosa Andrews/Paeonia lactiflora Pall	11896747
25	C_16_H_22_O_9_	358.13	Sweroside	403.1241; 357.1199; 195.0666; 179.0560; 125.0258	*Lonicera japonica* Thunb	10705325
29	C_17_H_20_O_9_	368.11	4-O-Feruloylquinic acid	193.0510; 173.0456; 134.0374	Phellodendron chinense C.K.Schneid	10636531
4	C_15_H_24_N_2_O	248.19	Sophoridine	249.1967; 180.1389; 152.1437; 150.1280	Sophora flavescens Aiton	8331481
54	C_27_H_34_O_11_	534.21	Arctiin	579.2163; 371.1535; 356.1311	TaraxacummongolicumHand.-Mazz	8006869
38	C_26_H_30_O_13_	550.17	Liquiritin apioside	549.1614; 255.0650; 135.0081; 119.0494	Glycyrrhiza uralensis Fisch. ex DC	7989242
22	C_16_H_18_O_9_	354.10	Cryptochlorogenic acid	353.0896; 19.0571; 173.0469; 135.0460	TaraxacummongolicumHand.-Mazz/*Lonicera japonica* Thunb	7822386
28	C_20_H_24_NO_4_+	342.17	Magnoflorine	342.1722; 297.1136; 265.0869; 58.0647	Phellodendron chinense C.K.Schneid	7400365
59	C_26_H_30_O_8_	470.19	Obaculactone	515.1899; 469.1859; 278.1287; 229.1219	Phellodendron chinense C.K.Schneid	7123925
10	C_16_H_18_O_9_	354.10	Neochlorogenic acid	353.0885; 191.0562; 179.0348; 135.0455	TaraxacummongolicumHand.-Mazz/*Lonicera japonica* Thunb	6939822
27	C_17_H_26_O_10_	390.15	Loganin	435.1498; 227.0916; 127.0396; 101.0240	*Lonicera japonica* Thunb	6815061
36	C_26_H_35_NO_11_	537.22	L-phenylalaninosecologanin	538.2330; 376.1783; 358.1672; 298.1451; 256.1353; 211.0975	*Lonicera japonica* Thunb	6737936
32	C_17_H_20_O_9_	368.11	5-O-Feruloylquinic acid	193.0499; 173.0451; 155.0343; 134.0367	Phellodendron chinense C.K.Schneid	6297611
20	C_23_H_28_O_12_	496.16	Oxypaeoniflora	495.1506; 333.0981; 177.0557; 137.0246	Paeonia× suffruticosa Andrews/Paeonia lactiflora Pall	6234230
40	C_27_H_30_O_16_	610.15	Rutin	609.1457; 301.0330; 300.0259; 271.0227	*Lonicera japonica* Thunb	5182657
19	C_17_H_20_O_9_	368.11	3-O-Feruloylquinicacid	367.1044; 193.0514; 134.0380	Phellodendron chinense C.K.Schneid	5096723
1	C_7_H_6_O_5_	170.02	Gallic acid	169.0147; 125.0245; 107.0132	Paeonia× suffruticosa Andrews/Paeonia lactiflora Pall	5082040
15	C_16_H_24_O_10_	376.14	8-Epiloganicacid	375.1269; 213.0758; 169.0862; 151.0762	*Lonicera japonica* Thunb	4619092
14	C_20_H_30_O_12_	462.17	Forsythoside E	461.1695; 315.1095; 205.0722; 135.0457	Forsythia suspensa (Thunb.) Vahl	4419465
34	C_22_H_26_O_10_	450.15	Forsythenside A	449.1444; 315.1074; 2530490; 193.0506; 175.0388	Forsythia suspensa (Thunb.) Vahl	3945241
41	C_30_H_32_O_15_	632.17	Galloylpaeoniflorin	631.1645; 613.1545; 491.1173; 399.0909; 313.0544; 169.0130	Paeonia× suffruticosa Andrews/Paeonia lactiflora Pall	3758978
18	C_16_H_24_O_10_	376.14	Loganic acid	375.1301; 195.0669; 169.0868; 151.0767	*Lonicera japonica* Thunb	3171568
50	C_27_H_34_O_11_	534.21	Phillyrin	579.2137; 533.2099; 371.1521; 356.1271; 207.0532; 161.0466	Forsythia suspensa (Thunb.) Vahl	3164862
57	C_42_H_62_O_17_	838.40	Licoricesaponin G2	837.3949; 351.0574; 193.0354	Glycyrrhiza uralensis Fisch. ex DC	3079077
58	C_42_H_62_O_17_	838.40	Uralsaponin U	837.3908; 351.0553; 193.0345	Glycyrrhiza uralensis Fisch. ex DC	3039823
8	C_13_H_12_O_9_	312.05	Caftaric acid	179.0347; 149.0089; 135.0444	TaraxacummongolicumHand.-Mazz	2460122
13	C_17_H_24_O_11_	404.13	Secoxyloganin	449.1284; 403.1223; 359.1331; 241.0706; 179.0554	*Lonicera japonica* Thunb	2447963
51	C_16_H_16_O_6_	304.09	Oxypeucedanin hydrate	305.1012; 203.0338; 159.0438; 147.0437	Angelica dahurica (Fisch.ex Hoffm.)Benth.et Hook.f	2401993
42	C_21_H_20_O_11_	448.10	Luteoloside	447.0919; 285.0387; 284.0309; 151.0025; 133.0280	*Lonicera japonica* Thunb	2356137
48	C_21_H_22_O_9_	418.13	Isoliquiritin	255.0670; 161.0265; 148.0164	Glycyrrhiza uralensis Fisch. ex DC	2001947
12	C_15_H_24_N_2_O_2_	264.18	Oxysophoridine	265.1915; 247.1808; 205.1336; 177.1384	Sophora flavescens Aiton	1774855
11	C_10_H_10_O_5_	210.05	(p-Hydroxybenzyl)malonic acid	209.0558; 121.0662; 93.0350	Glycyrrhiza uralensis Fisch. ex DC	1597260
53	C_30_H_32_O_13_	600.18	Benzoyloxypaeoniflorin	599.1731; 477.1331; 385.0885; 165.0552; 137.0239; 121.0288	Paeonia× suffruticosa Andrews/Paeonia lactiflora Pall	1538968
49	C_26_H_30_O_13_	550.17	Isoliquiritin apioside	549.1605; 255.0640; 135.0086; 119.0499	Glycyrrhiza uralensis Fisch. ex DC	1316350
9	C_7_H_6_O_3_	138.03	p-Hydroxybenzoic acid	137.0237; 108.0218; 92.0272	Momordica cochinensis (Lour.)Spreng	898282
16	C_15_H_14_O_6_	290.08	Catechin	289.0712; 245.0825; 203.0730; 151.0425; 109.0300	TaraxacummongolicumHand.-Mazz	482601
56	C_30_H_32_O_12_	584.19	Benzoylpaeoniflorin	583.1811; 431.1336; 165.0553; 121.0293	Paeonia× suffruticosa Andrews/Paeonia lactiflora Pall	629.189
55	C_17_H_18_O_7_	334.11	Byakangelicin	233.0438; 231.0283; 218.0211; 203.0332; 175.0390	Angelica dahurica (Fisch.ex Hoffm.)Benth.et Hook.f	317.1019

Metabolites compositional identification using UPLC-Q-TOF/MS revealed 60 metabolites, 7 metabolites from Phellodendron chinense C.K Schneid, 6 metabolites from Forsythia suspensa (Thunb.) Vahl, 18 metabolites from *Lonicera japonica* Thunb, 7 metabolites from Paeonia × suffruticosa Andrews, with 7 metabolites from Paeonia lactiflora Pall, with 8 metabolites from Glycyrrhiza uralensis Fisch. ex DC, with 1 metabolite from Momordica cochinensis (Lour.) Spreng, 10 metabolites from TaraxacummongolicumHand.-Mazz, 2 metabolites from Angelica dahurica (Fisch. ex Hoffm.) Benth. et Hook. f, 7 metabolites from Sophora flavescens Aiton. Higher levels of Berberine from Phellodendron chinense C.K. Schneid, Forsythoside A from Forsythia suspensa (Thunb.) Vahl, Paeoniflorin from Paeonia × suffruticosa Andrews/Paeonia lactiflora Pall, Vogeloside from *Lonicera japonica* Thunb, Secologanin from *Lonicera japonica* Thunb, Piscidic acid from Sophora flavescens Aiton, Isochlorogenicacid C from TaraxacummongolicumHand.-Mazz/*Lonicera japonica* Thunb, and Matrine from Sophora flavescens Aiton (Shang et al., 2011).

### 3.2 Ferroptosis levels in VU tissues were higher than those in normal tissues

**FIGURE 2:**
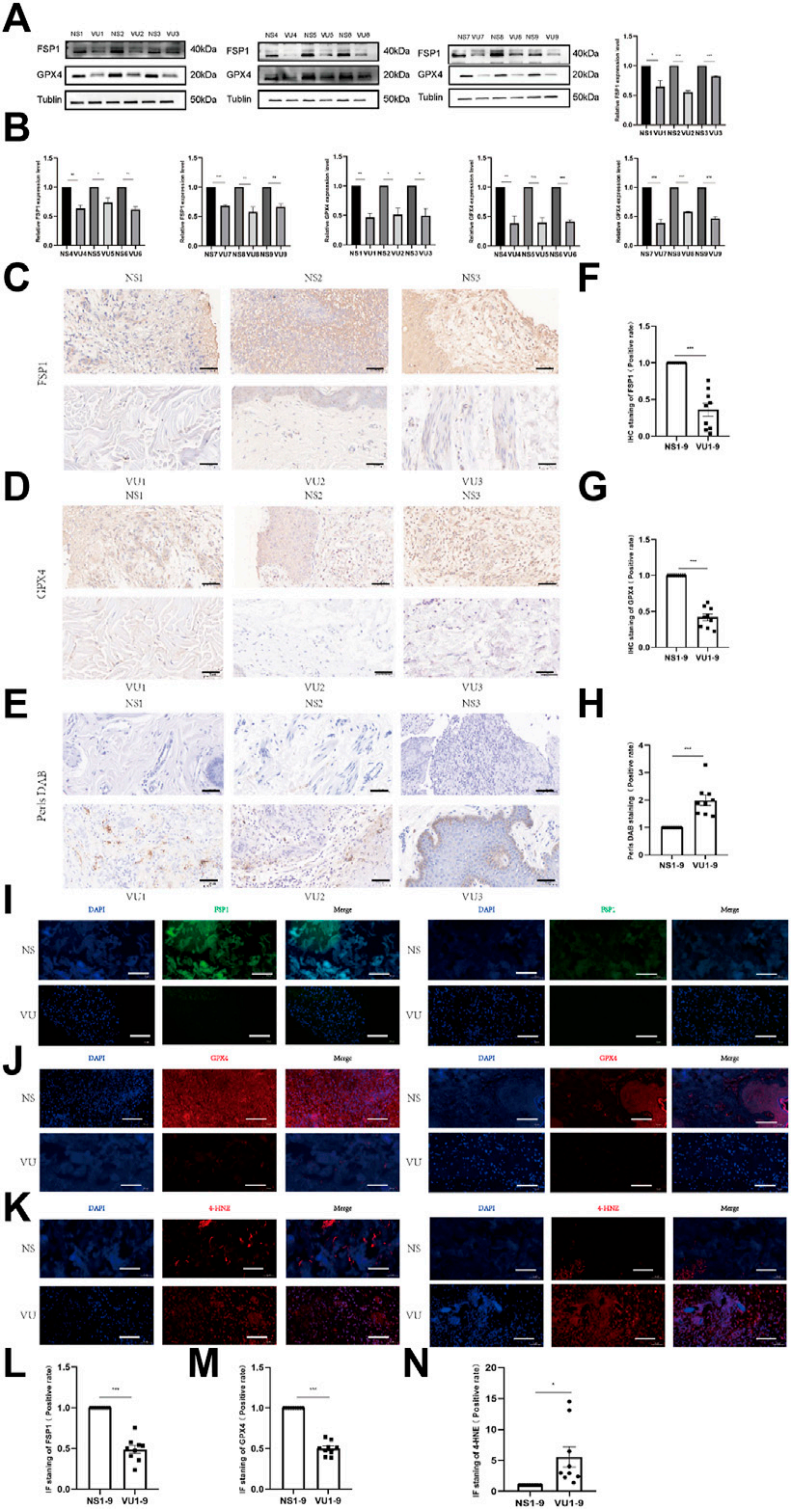
Differences in expression between venous ulcer (VU) tissues and normal tissues. **(A)** Western blot analysis of FSP1 and GPX4 in 9 cases of VU tissue and normal skin tissue. **(B)** Quantification of the protein band intensities in **(A)**. The data were normalized by lane normalization factor (LNF). **(C)** IHC staining of FSP1 in VU tissue and normal tissue sections. Scale bar = 50 μm. **(D)** IHC staining of GPX4 in VU tissue and normal tissue sections. Scale bar = 50 μm. **(E)** DAB-Prussian blue staining of ferrous ions in VU tissue and normal tissue sections. Scale bar = 50 μm. **(F)** Quantitative analysis of FSP1 expression according to IHC staining in **(C)**. **(G)** Quantitative analysis of GPX4 expression according to IHC staining in **(D)**. **(H)** Quantitative analysis of ferrous ion expression according to DAB-Prussian Blue staining in **(E)**. **(I)** Confocal IF of FSP1 (green) and DAPI (blue). Scale bar = 50 μm. **(J)** Confocal IF of GPX4 (red) and DAPI (blue). Scale bar = 50 μm. **(K)** Confocal IF of 4-HNE (red) and DAPI (blue). Scale bar = 50 μm. **(L)** Quantitative analysis of FSP1 expression according to IF staining in **(I)**. **(M)** Quantitative analysis of GPX4 expression according to IF staining in **(J)**. **(N)** Quantitative analysis of 4-HNE expression according to IF staining in **(K)**. The experiments were performed in triplicate. The data are presented as the means ± SDs, and significant differences were evaluated using unpaired t tests. *p < 0.05, **p < 0.01 and ***p < 0.005.

We collected normal tissues and ulcer tissues from 9 VU patients, and Western blotting was performed to measure tissue protein expression. As shown in Figures 2A, B, the expression of FSP1 and GPX4 in ulcer tissues was significantly lower than that in normal tissues. Prussian blue staining results are shown in Figures 2E, H. Prussian blue staining showed that the iron ion content of ulcerated tissues was higher than that of normal tissues. The expression levels of FSP1 (Figures 2C, F) and GPX4 (Figures 2D, G) in ulcerated tissues were lower than those in normal tissues according to IHC. IF also showed that the expression levels of FSP1 (Figures 2I, L) and GPX4 (Figures 2J, M) in ulcerated tissues were lower than those in normal tissues. The 4-HNE lipid peroxidation level assay showed that the lipid peroxidation level in ulcerated tissues was significantly higher than that in normal tissues (Figures 2K, N).

### 3.3 Ferroptosis levels in VU tissues were decreased after DDL treatment

**FIGURE 3:**
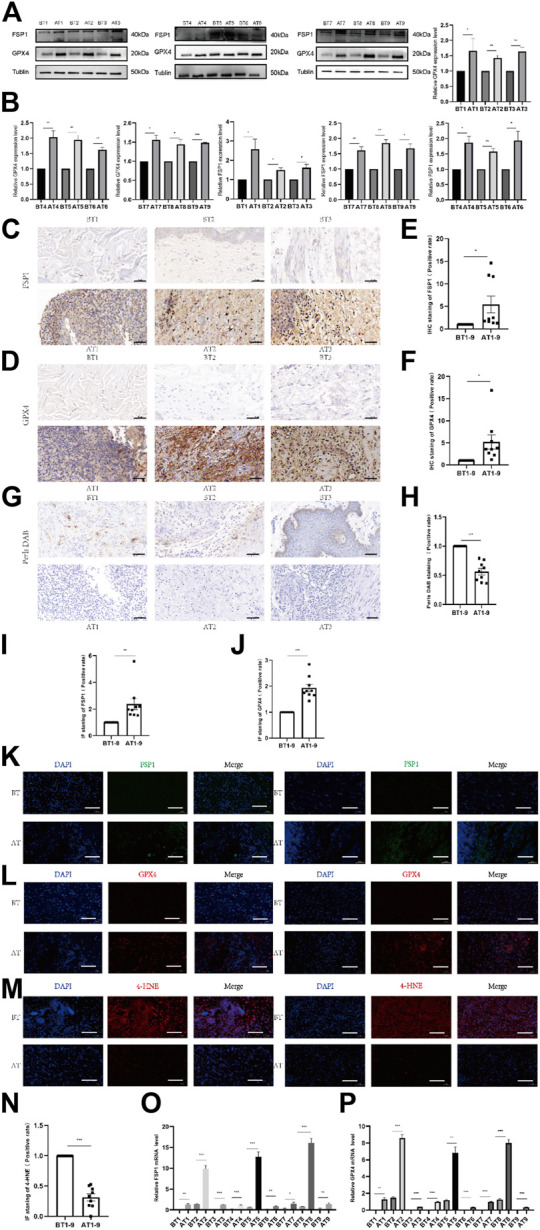
Comparison of tissue expression before and after DDL treatment of VUs. **(A)** Western blot analysis of FSP1 and GPX4 before and after tissue in 9 VUs treated with DDL. **(B)** Quantification of the protein band intensities in **(A)**. The data were normalized to LNF. **(C)** IHC staining of FSP1 before and after DDL treatment of VU sections. Scale bar = 50 μm. **(D)** IHC staining of GPX4 before and after DDL treatment of VU sections. Scale bar = 50 μm. **(E)** Quantitative analysis of FSP1 expression according to IHC staining in **(C)**. **(F)** Quantitative analysis of GPX4 expression according to IHC staining in **(D)**. **(G)** DAB-Prussian blue staining of ferrous ions before and after DDL treatment of VU sections. Scale bar = 50 μm. **(H)** Quantitative analysis of ferrous ion expression according to DAB-Prussian blue staining in **(G)**. **(K)** Confocal IF of FSP1 (green) and DAPI (blue). Scale bar = 50 μm. **(L)** Confocal IF of GPX4 (red) and DAPI (blue). Scale bar = 50 μm. **(M)** Confocal IF of 4-HNE (red) and DAPI (blue). Scale bar = 50 μm. **(I)** Quantitative analysis of FSP1 expression according to IF staining in **(K)**. **(J)** Quantitative analysis of GPX4 expression according to IF staining in **(L)**. **(N)** Quantitative analysis of 4-HNE expression according to IF staining in **(M)**. **(O)** qPCR analysis of FSP1 mRNA levels before and after DDL treatment of VUs. **(P)** qPCR analysis of GPX4 mRNA levels before and after DDL treatment of VUs. The experiments were performed in triplicate. The data are presented as the means ± SDs, and significant differences were evaluated using unpaired t tests. *p < 0.05, **p < 0.01 and ***p < 0.005.

We collected VU tissues before and after DDL treatment, and the protein expression of FSP1 and GPX4 in VU tissues after DDL treatment was significantly higher than that in VU tissues before treatment (Figures 3A, B). Prussian blue staining showed lower iron ion contents in VU tissues after treatment than in VU tissues before treatment (Figures 3G, H). The expression levels of FSP1 (Figures 3C, E) and GPX4 (Figures 3D, F) in VU tissues after treatment were higher than those in VU tissues before treatment according to IHC, and IF also showed lower expression levels of FSP1 (Figures 3I, K) and GPX4 (Figures 3J, L) in VU tissues before treatment than in VU tissues after treatment. The 4-HNE lipid peroxidation level assay showed that the lipid peroxidation level in VU tissues before treatment was significantly higher than that in VU tissues after treatment (Figures 3M, N). At the RNA level, FSP1 (Figure 3O) and GPX4 (Figure 3P) were significantly higher in VU tissues after DDL treatment than in VU tissues before treatment.

### 3.4 Hemin-induced ferroptosis occurred in HFFs

**FIGURE 4:**
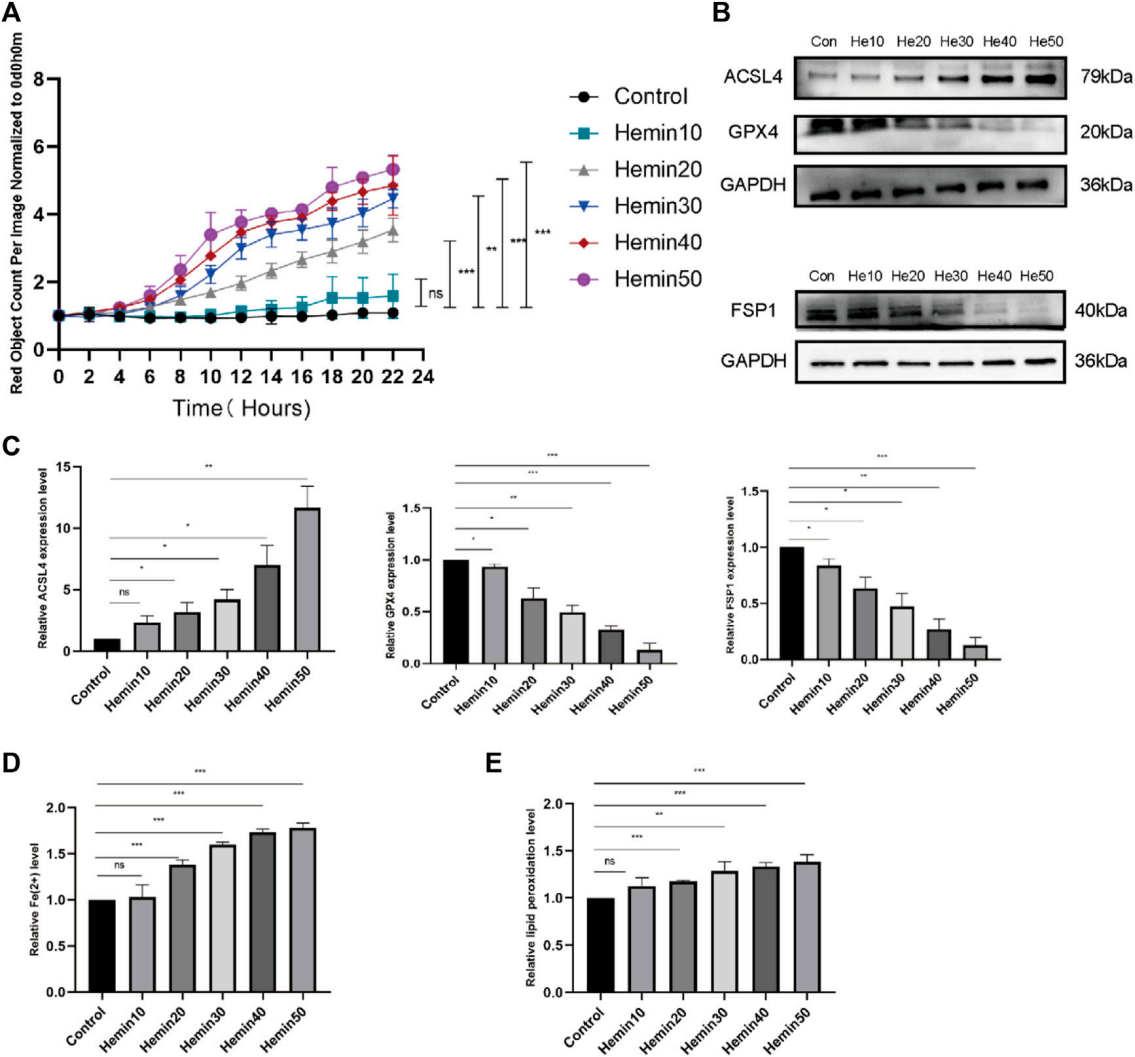
Different concentrations of hemin affected fibroblast activity and caused elevated expression of ACSL4 and decreased expression of FSP1 and GPX4. **(A)** DiYO™-3 working solution was added to DMEM containing 15% FBS at a dilution of 1:8000 to label dead cells with red staining. The number of dead cells was measured with normalization to hour 0 and calculated using IncuCyte (Essen BioScience). **(B)** Western blot analysis of ACSL4, FSP1, and GPX4 after treatment with different concentrations of hemin. **(C)** Quantification of the protein band intensities in **(B)**. The data were normalized to LNF. **(D)** HFFs were treated with 0, 10, 20, 30, 40, or 50 μmol/L hemin, and changes in intracellular divalent iron ion content were detected by flow cytometry through the PE channel. **(E)** HFFs were treated with 0, 10, 20, 30, 40, or 50 μmol/L hemin and assayed by flow cytometry through the FITC and PE channels, with the ratio of FITC to PE representing the intracellular lipid peroxidation level. The experiments were performed in triplicate. The data are presented as the means ± SDs, and significant differences were evaluated using unpaired t tests. *p < 0.05, **p < 0.01, ***p < 0.005 and ns, not significant.

The pathogenesis of VUs mainly involves hemoglobin extravasation due to impaired venous return, so we established a model of VUs with hemin. Cell survival changed as the concentration of hemin changed; that is, cell death increased with increasing hemin concentrations at concentrations that could be observed in the cell field with the IncuCyte S3 program (Figure 4A). We also measured the expression of acyl-CoA synthetase long-chain family member 4 (ACSL4), FSP1, and GPX4 in the cells by protein immunoblotting, and we found that the expression of ACSL4 gradually increased, while that of FSP1 and GPX4 gradually decreased, with increasing hemin concentrations (Figures 4B, C). Flow cytometry was performed to measure the contents of divalent iron (Fe^2+^) after HFFs were treated with different concentrations of hemin (0 μmol/L, 10 μmol/L, 20 μmol/L, 30 μmol/L, 40 μmol/L and 50 μmol/L). As shown in Figure 4D, the intracellular divalent iron content increased with increasing hemin concentrations. We examined the lipid peroxidation levels in the cells, and the intracellular lipid peroxidation levels tended to decrease with increasing hemin concentrations (Figure 4E). Although hemin caused these changes, we are not sure whether it actually caused ferroptosis; it is possible that the cells remained at the lipid peroxidation stage and that ferroptosis did not occur. For this reason, we performed further verification experiments.

**FIGURE 5:**
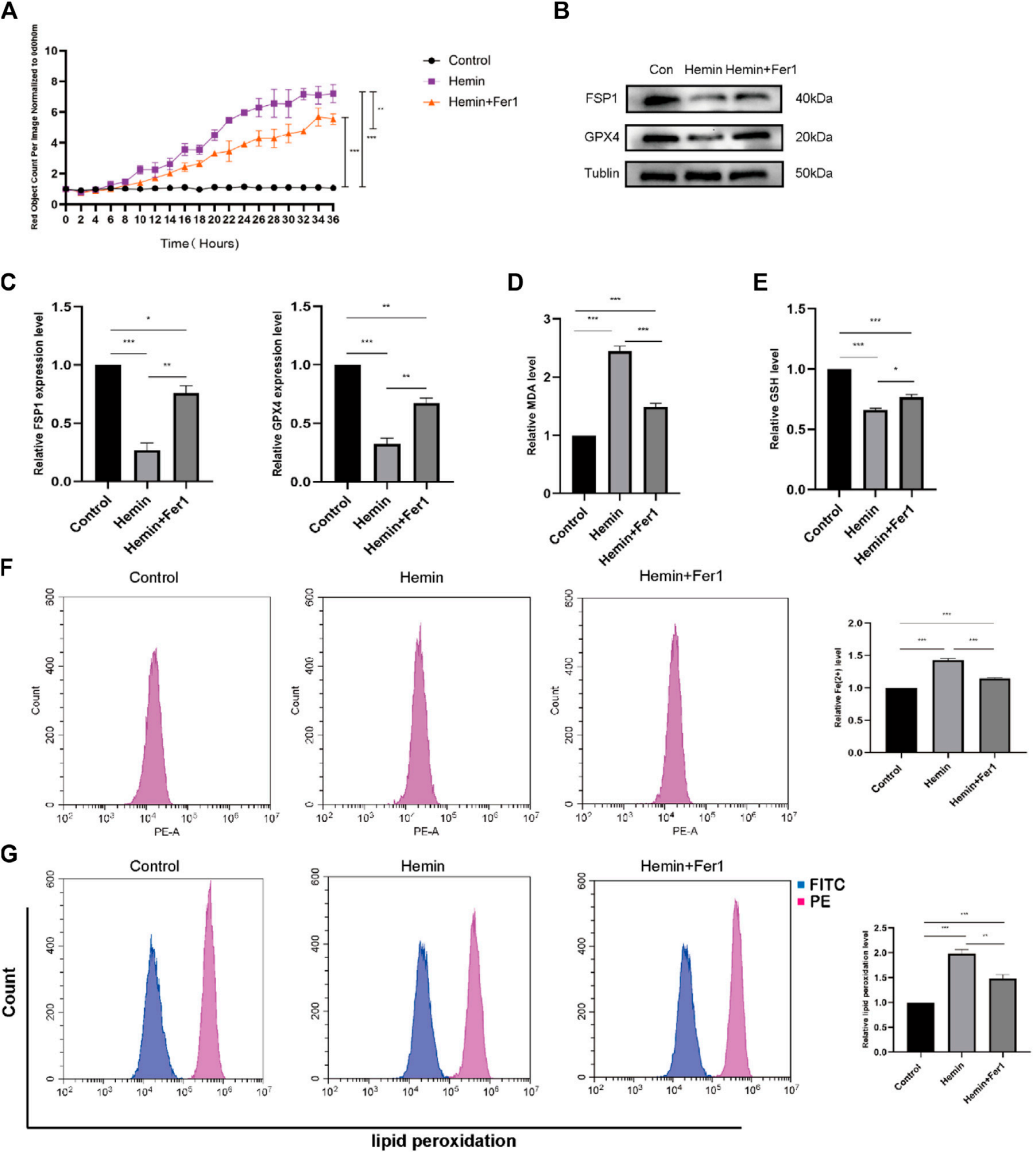
Fer-1 rescued hemin-induced ferroptosis. **(A)** As in the previously described method, DiYO™-3 was used to label HFFs. The number of dead cells was normalized to that at hour 0 and calculated using IncuCyte (Essen BioScience). **(B)** Western blot analysis of FSP1 and GPX4. **(C)** Quantification of the protein band intensities in **(B)**. The data were normalized to LNF. **(D)** Malondialdehyde (MDA) levels were measured. **(E)** GSH levels were measured. **(F)** HFFs were treated with hemin and hemin + Fer-1, and changes in intracellular divalent iron ion content were detected by flow cytometry through the PE channel. **(G)** HFFs were treated with hemin and hemin + Fer-1 and assayed by flow cytometry through the FITC and PE channels, with the ratio of FITC to PE representing the intracellular lipid peroxidation level. The experiments were performed in triplicate. The data are presented as the means ± SDs, and significant differences were evaluated using an unpaired *t*-test. *p < 0.05, **p < 0.01, ***p < 0.005.

We selected the optimal concentration of hemin (50 μmol/L) and performed a set of experiments that involved the addition of Fer-1. According to the IncuCyte S3 program assay, the cell mortality rate of cells that were treated with Fer-1 was lower than that of cells that were treated with hemin (Figure 5A), suggesting that Fer-1 might reverse the effects of hemin. For this reason, we measured the levels of ACSL4. The levels of ACSL4 in the Fer-1 group were lower than those in the hemin group, and the levels of GPX4 were higher than those in the hemin group (Figures 5B, C). As shown in Figure 5F, the divalent iron levels in the Fer-1 group were lower than those in the hemin group. Additionally, we found that the lipid peroxidation levels in the Fer-1 group were lower than those in the hemin group (Figure 5G) according to flow cytometry. In addition, we measured the GSH and MDA levels. As shown in Figure 5E, the GSH levels in the Fer-1 group were higher than those in the hemin group; as shown in Figure 5D, the MDA levels in the Fer-1 group were lower than those in the hemin group. The experimental results revealed that hemin may lead to substantial cellular ferroptosis.

### 3.5 Inhibitory effect of DDL on hemin-induced ferroptosis

**FIGURE 6:**
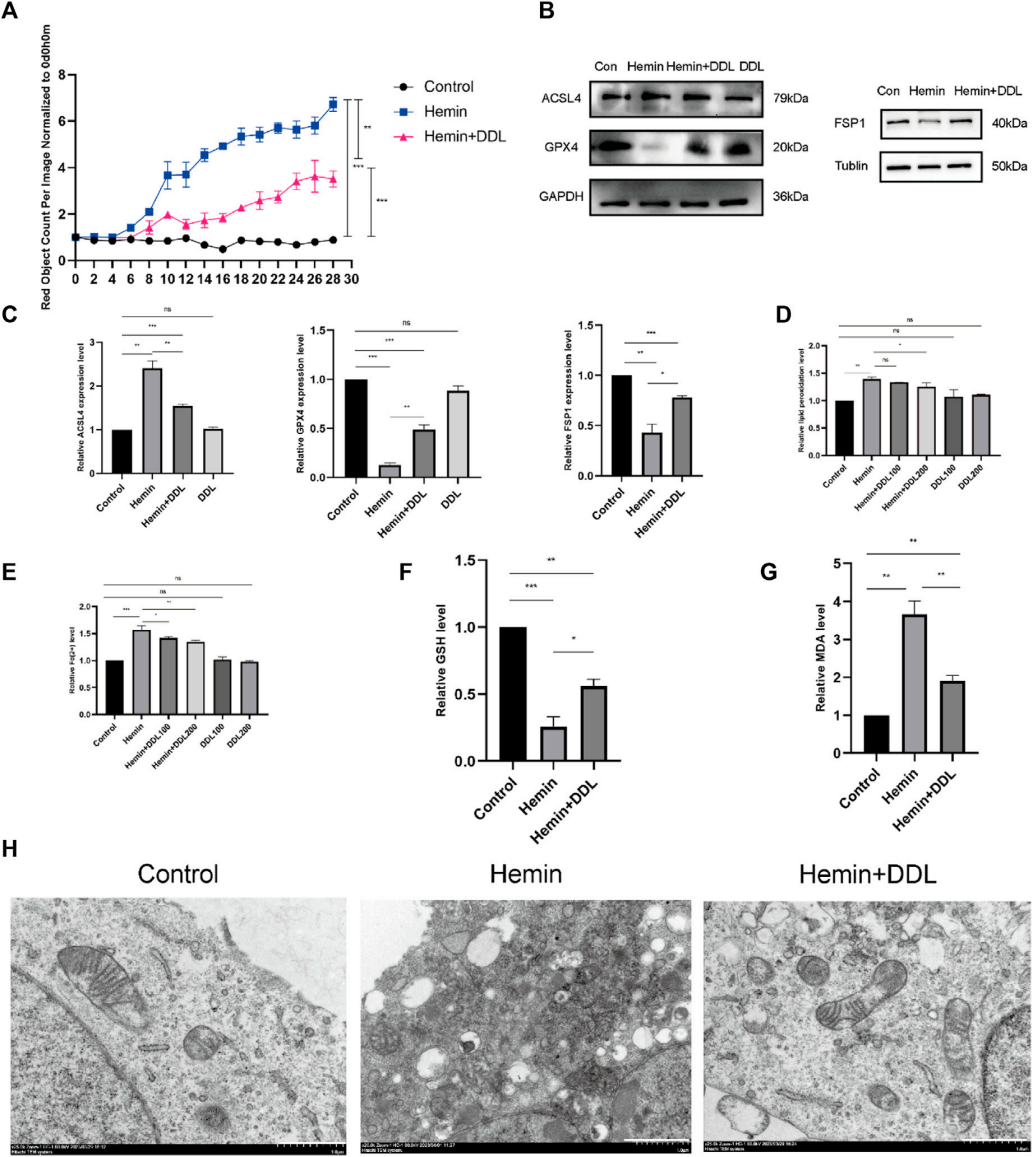
DDL inhibits hemin-induced ferroptosis. **(A)** As in the previously described method, DiYO™-3 was used to label HFFs. The number of dead cells was normalized to that at hour 0 and calculated using IncuCyte (Essen BioScience). **(B)** Western blot analysis of ACSL4, FSP1 and GPX4. **(C)** Quantification of the protein band intensities in **(B)**. The data were normalized to LNF. **(D)** HFFs were treated with hemin and hemin + DDL and assayed by flow cytometry through the FITC and PE channels, with the ratio of FITC to PE representing the intracellular lipid peroxidation level. **(E)** HFFs were treated with hemin and hemin + DDL, and changes in the intracellular divalent iron ion content were detected by flow cytometry through the PE channel. **(F)** Measurement of glutathione (GSH) levels. **(G)** Transmission electron microscopy observation of mitochondrial morphology. Hemin-treated cells (14 h) partially presented a necrotic phenotype with loss of plasma membrane integrity and disintegration of organelles, and the mitochondrial volume was significantly reduced. Nonhematoxylin-treated cells showed intact plasma membranes and larger mitochondria; hematoxylin- and DDL-treated mitochondria were also reduced in size but not as markedly as those treated with hemin alone. Scale bar = 1000 nm. **(H)** Measurement of malondialdehyde (MDA) levels. The experiments were performed in triplicate. The data are presented as the means ± SDs, and significant differences were evaluated using unpaired t tests. *p < 0.05, **p < 0.01, ***p < 0.005.

We seeded cells in 96-well plates and observed them with the IncuCyte S3 program, and the results are shown in Figure 6A. The cell mortality rate of the hemin + DDL group was significantly lower than that of the hemin group. We measured the expression of ACSL4, FSP1 and GPX4 in the different groups by protein immunoblotting and found that the expression of ACSL4 in the hemin + DDL group was lower and the expression of FSP1 and GPX4 was higher than those in the hemin group (Figures 6B,C). Flow cytometry was used to measure the divalent iron levels in the different groups, and the results showed that the divalent iron levels were lower in the hemin + DDL group than in the hemin group (Figure 6E). Additionally, the hemin + DDL group had lower lipid peroxidation levels than the hemin group (Figure 6D). In addition, we also performed TEM experiments to observe the mitochondrial morphology of the different groups, and the results are shown in Figure 6H. Mitochondrial wrinkling was worse in the hemin group than in the hemin + DDL group, and the reduction in mitochondrial volume was most obvious in the hemin group. In addition, we measured the GSH and MDA levels. As shown in Figure 6F, the GSH levels in the hemin + DDL group were higher than those in the hemin group; as shown in Figure 6G, the MDA levels in the hemin + DDL group were lower than those in the hemin group.

### 3.6 DDL inhibited hemin-induced ferroptosis via the CoQ-FSP1 axis

**FIGURE 7:**
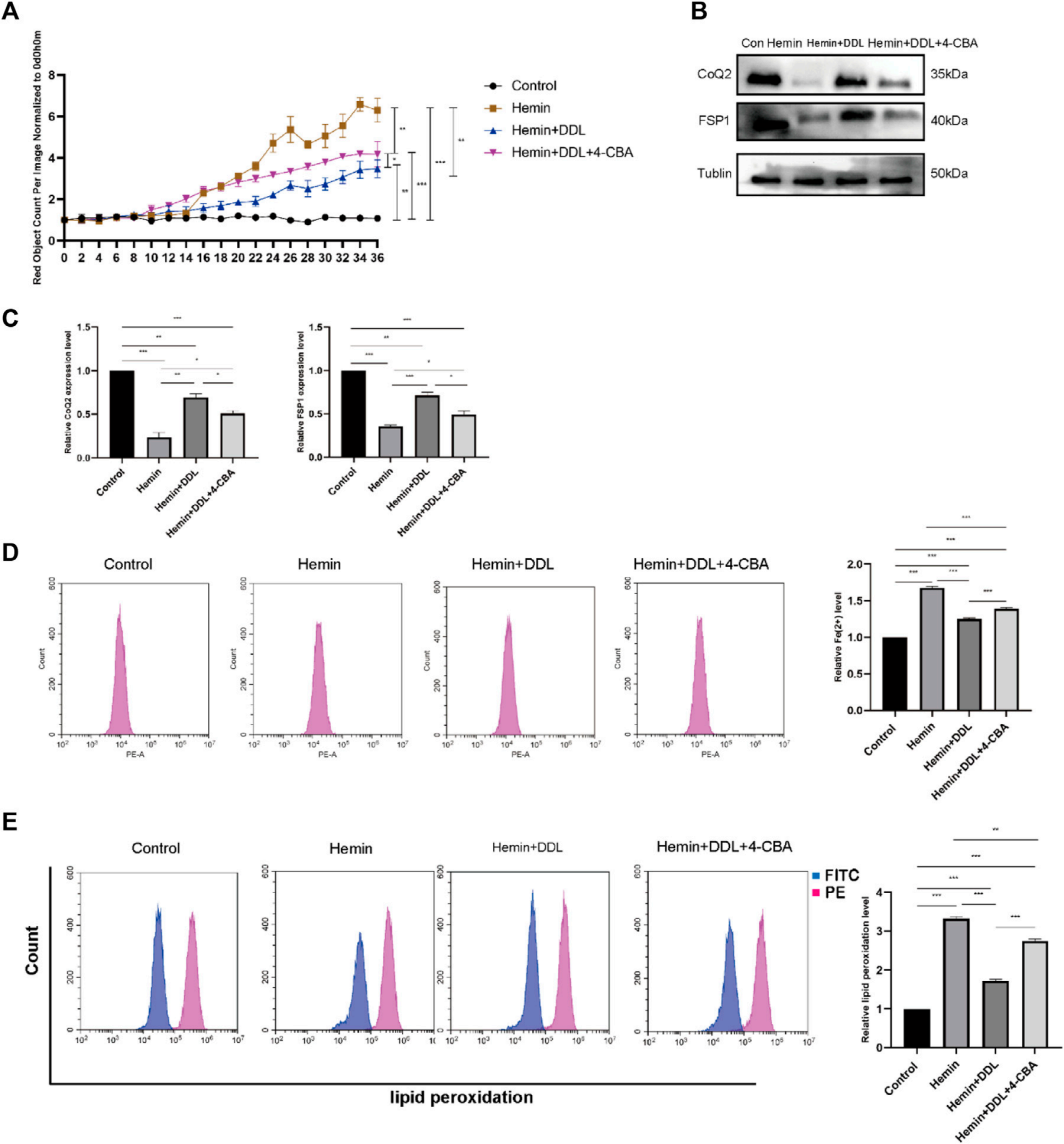
CoQ2 inhibition affected DDL exertion. **(A)** DiYO™-3 was used to label HFFs. The number of dead cells was normalized to that at hour 0 and calculated using IncuCyte (Essen BioScience). **(B)** Western blot analysis of CoQ2 and FSP1. **(C)** Quantification of the protein band intensities in **(B).** The data were normalized to LNF. **(D)** HFFs were treated with hemin, hemin + DDL, and hemin + DDL+4-CBA, and changes in the intracellular divalent iron ion content were detected by flow cytometry through the PE channel. **(E)** HFFs were treated with hemin, hemin + DDL, and hemin + DDL+4-CBA and assayed by flow cytometry through the FITC and PE channels, with the ratio of FITC to PE representing the intracellular lipid peroxidation level. The experiments were performed in triplicate. The data are presented as the means ± SDs, and significant differences were evaluated using unpaired t tests. *p < 0.05, **p < 0.01, ***p < 0.005.

We inhibited CoQ using the CoQ inhibitor 4-CBA and divided the cells into four groups. We used the IncuCyte S3 program to measure cell viability and found that CoQ inhibition influenced the effect of DDL. As shown in Figure 7A, the cell mortality rates of both the hemin + DDL group and hemin + DDL+4-CBA group were lower than that of the hemin group. However, the cell mortality rate in the hemin + DDL+4-CBA group was higher than that in the hemin + DDL group, and the cell mortality rate in all three groups was higher than that in the control group. We measured the expression of FSP1 and CoQ in the different groups by protein immunoblotting, and we found that the protein levels of FSP1 and CoQ in the hemin + DDL+4-CBA group were higher than those in the hemin group but lower than those in the hemin + DDL group (Figures 7B, C); the protein levels of FSP1 and CoQ in all three groups were lower than those in the control group. The levels of divalent iron in the different groups were measured by flow cytometry, and they were lower in the hemin + DDL+4-CBA group than in the hemin group but higher than in the hemin + DDL group (Figure 7D); additionally, the levels of divalent iron were higher in all three groups than in the control group. Finally, the lipid peroxidation levels were measured. The levels in the hemin + DDL+4-CBA group were lower than those in the hemin group but higher than those in the hemin + DDL group (Figure 7E), and the levels of lipid peroxidation in all three groups were higher than those in the control group.

**FIGURE 8:**
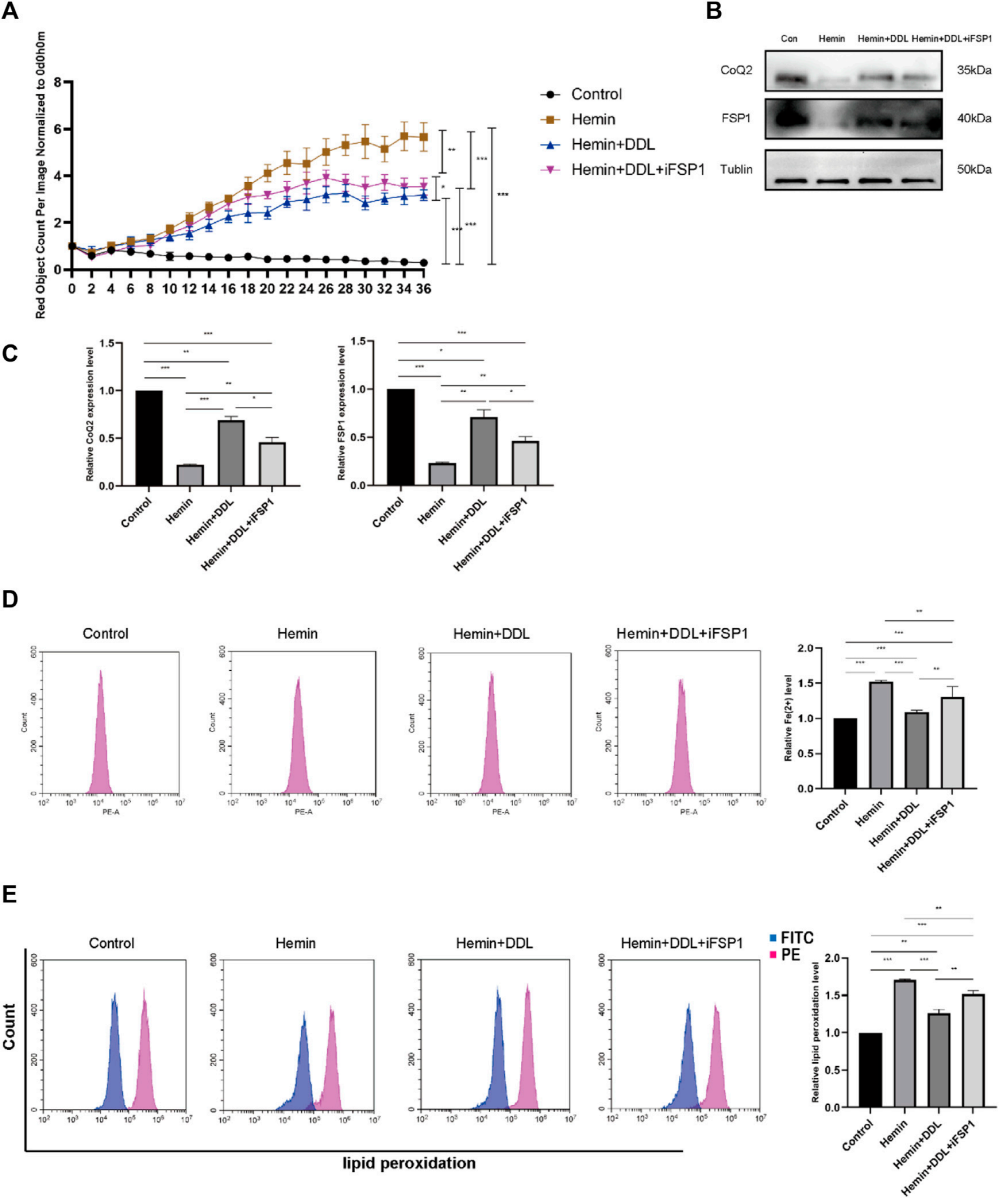
Inhibition of FSP1 affected the exertion of DDL. **(A)** DiYO™-3 was used to label HFFs. The number of dead cells was normalized to that at hour 0 and calculated using IncuCyte (Essen BioScience). **(B)** Western blot analysis of CoQ2 and FSP1. **(C)** Quantification of the protein band intensities in **(B)**. The data were normalized to LNF. **(D)** HFFs were treated with hemin, hemin + DDL, and hemin + DDL + iFSP1, and changes in the intracellular divalent iron ion content were detected by flow cytometry through the PE channel. **(E)** HFFs were treated with hemin, hemin + DDL, and hemin + DDL + iFSP1 and assayed by flow cytometry through the FITC and PE channels, with the ratio of FITC to PE representing the intracellular lipid peroxidation level. The experiments were performed in triplicate. The data are presented as the means ± SDs, and significant differences were evaluated using an unpaired *t*-test. *p < 0.05, **p < 0.01, ***p < 0.005.

We used the FSP1 inhibitor iFSP1 to inhibit FSP1 and divided the cells into 4 groups, as shown in Figure 8A. We used the IncuCyte S3 program to determine cell viability and found that FSP1 inhibition influenced the effect of DDL; that is, the cell mortality rates in the hemin + DDL group and hemin + DDL + iFSP1 group were lower than that in the hemin group, but the cell mortality rate in the hemin + DDL + iFSP1 group was higher than that in the hemin + DDL group. Additionally, the cell mortality rates in all three groups were higher than that in the control group. We measured the expression of FSP1 and CoQ in the different groups by protein immunoblotting, and we found that the FSP1 protein levels were higher in the hemin + DDL + iFSP1 group than in the hemin group but lower than in the hemin + DDL group (Figures 8B,C); additionally, the FSP1 protein levels in all three groups were lower than those in the control group. The CoQ levels were higher in the hemin + DDL + iFSP1 group than in the hemin group but were not significantly different from those in the hemin + DDL group; additionally, the CoQ levels in all three groups were lower than those in the control group. The levels of divalent iron in the different groups were measured by flow cytometry, and they were lower in the hemin + DDL + iFSP1 group than in the hemin group but higher than in the hemin + DDL group (Figure 8D). Additionally, the levels of divalent iron in all three groups were higher than those in the control group. Finally, lipid peroxidation levels were measured, and the levels in the hemin + DDL + iFSP1 group were lower than those in the hemin group but higher than those in the hemin + DDL group; additionally, the levels of lipid peroxidation in all three groups were higher than those in the control group (Figure 8E).

### 3.7 DDL promoted VU healing by inhibiting ferroptosis through the CoQ-FSP1 axis

**FIGURE 9:**
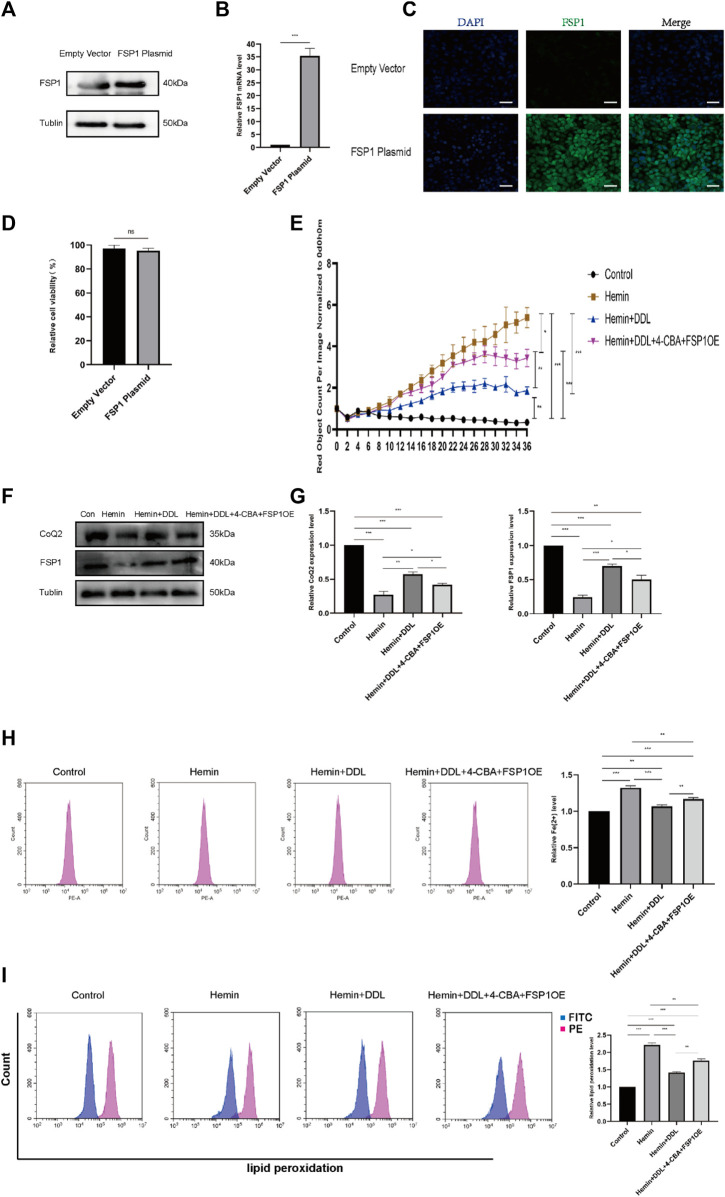
Overexpression of FSP1 and inhibition of CoQ2 also affected DDL function. **(A)** Western blots showing FSP1 overexpression in the FSP1 plasmid *versus* empty vector. **(B)** qPCR analysis of FSP1 mRNA levels. **(C)** IF results showing FSP1 overexpression by the FSP1 plasmid compared with the empty vector. Scale bar = 50 μm. **(D)** Cell viability was determined by CCK-8 assay and compared between the FSP1 plasmid group and the empty vector group. **(E)** DiYO™-3 was used to label HFFs. The number of dead cells was normalized to that at hour 0 and calculated using IncuCyte (Essen BioScience). **(F)** Western blot analysis of CoQ2 and FSP1. **(G)** Quantification of the protein band intensities in **(F)**. The data were normalized to LNF. **(H)** HFFs were treated with hemin, hemin + DDL, and hemin + DDL+4-CBA + FSP1OE, and changes in the intracellular divalent iron ion content were detected by flow cytometry through the PE channel. **(I)** HFFs were treated with hemin, hemin + DDL, and hemin + DDL+4-CBA + FSP1OE and assayed by flow cytometry through the FITC and PE channels, with the ratio of FITC to PE representing the intracellular lipid peroxidation level. The experiments were performed in triplicate. The data are presented as the means ± SDs, and significant differences were evaluated using unpaired t tests. *p < 0.05, **p < 0.01, ***p < 0.005 and ns, not significant.

Based on the results of the previous experiment, we conducted additional experiments. Combined with our experiments on the CoQ-FSP1 axis, which revealed that inhibition of either CoQ or FSP1 alone influenced the effect of DDL, we speculate that DDL may inhibit hemin-induced ferroptosis through the CoQ-FSP1 axis and that DDL may promote wound healing of VUs by inhibiting ferroptosis through the CoQ-FSP1 axis. We overexpressed FSP1 via transfection, and Western blotting and qPCR analyses with approximately the same numbers of cells showed that FSP1 protein expression and mRNA transcription were significantly higher in the FSP1-overexpressing group than in the control group (Figures 9A, B). Immunofluorescence results revealed that FSP1 was expressed in both the FSP1-overexpressing and control groups, but the fluorescence intensity was stronger in the FSP1-overexpressing group (Figure 9C). The results of the cell proliferation and cell viability assays showed that there was no difference in cell proliferation between the two groups (Figure 9D).

Based on previous experiments, we conducted experiments with FSP1-overexpressing cells. We treated FSP1-overexpressing cells with hemin and DDL, inhibited CoQ by treatment with the CoQ inhibitor 4-CBA, and established a hemin + DDL+4-CBA + FSP1OE group; ultimately, the cells were divided into 4 groups, as shown in Figure 9E. The IncuCyte S3 program was used to assess cell activity, and the results showed that simultaneous FSP1 overexpression and CoQ inhibition influenced the effect of DDL. The cell mortality rates of the hemin + DDL and hemin + DDL+4-CBA + FSP1OE groups were lower than that of the hemin group, but the cell mortality rate of the hemin + DDL+4-CBA + FSP1OE group was higher than that of the hemin + DDL group; additionally, the cell mortality rates of all three groups were higher than that of the control group. We measured the expression of FSP1 and CoQ in the different groups by protein immunoblotting, and we found that the FSP1 and CoQ protein levels in the hemin + DDL+4-CBA + FSP1OE group were higher than those in the hemin group but lower than those in the hemin + DDL group; additionally, the FSP1 and CoQ protein levels in all three groups were lower than those in the control group (Figures 9F, G). The levels of divalent iron in the different groups were measured by flow cytometry, and they were lower in the hemin + DDL+4-CBA + FSP1OE group than in the hemin group but higher than in the hemin + DDL group; additionally, the levels of divalent iron in all three groups were higher than those in the control group (Figure 9H). Finally, the lipid peroxidation levels in the hemin + DDL+4-CBA + FSP1OE group were lower than those in the hemin group but higher than those in the hemin + DDL group; additionally, the lipid peroxidation levels in all three groups were higher than those in the control group (Figure 9I). These results suggest that DDL inhibited hemin-induced ferroptosis through the CoQ-FSP1 axis and suppressed ferroptosis in VUs, promoting wound healing.

## 4 Discussion

The incidence of VUs in the lower extremities has been increasing in recent years, and these ulcers are persistent and prone to recurrence, which seriously affects the quality of life of patients and causes great challenges for clinical treatment (Zhu et al., 2011). The occurrence of CVI is related to an increase in venous pressure due to insufficient venous reflux power and venous structural disorders, and venous hypertension leads to the extravasation of iron-containing hemoglobin and increases in iron deposition. The skin of VU patients has dark brown pigmentation, some patients have even darker skin, and Prussian blue staining indicates that the iron ion content is significantly higher than that of surrounding normal skin (Gupta et al., 2017). The dark brown pigmentation was significantly ameliorated after DDL treatment, and the iron ion content was also lower than that before treatment. By collecting ulcerated tissue samples and measuring FSP1 levels, CoQ levels, GPX4 levels, lipid peroxidation levels, Fe^2+^ levels, and 4-HNE levels, we found for the first time that intense ferroptosis occurs in VU tissues.

DDL has been clinically proven to be effective, safe and reliable in the treatment of VUs for more than 40 years, but its molecular mechanism is still unclear. TCM compounds have remarkable therapeutic effects on a wide range of diseases. Their complex composition, multifaceted therapeutic targets and sophisticated treatment modalities pose a great challenge to the combination therapy. However, the limitations of the DDL in our study were the lack of some medical pharmacological data, dose-effect relationship of metabolites, and pharmacological diversity of metabolites in each formulation. Therefore, the generation of new metabolites by DDL is only a hypothesis and further pharmacological experiments are needed to verify this finding. However, these these 60 metabolites we screened for the time being did not reveal any single botanical drug that does not belong to the DDL. but due to the small number of metabolites we screened and the need to expand the number of screenings at a later stage, it is not possible to demonstrate that botanical drug interactions in the DDL do not produce new metabolites, and that this new metabolite does not belong to any single botanical drug in the DDL.

Among the metabolites screened by DDL, Berberine was the most abundant. Berberine has been found to have significant antibacterial and anti-inflammatory effects (Sadeghi et al., 2024), as well as antioxidant and lipid metabolism regulation (Akhzari et al., 2024). Most botanical drugs that are included in this formula are rich in flavonoids (Sun et al., 2023), examples include Forsythoside, Catechin, Paeoniflorin and Luteoloside, among many other metabolites, which are a large class of plant-derived metabolites that perform multiple biological functions; flavonoids exert therapeutic effects on skin diseases and have strong antioxidant effects (Carvalho et al., 2021). Modern studies on some drugs suggest that they exert antibacterial and antioxidant effects, but the effects of any particular Chinese medicine are not simply the sum of the effects of the individual metabolites; rather, Chinese medicines exert their effects via complex processes of biological and chemical changes.

Although the efficacy of DDL in the treatment of lower limb VUs has been studied in the early stage, studies on the mechanism underlying its antibacterial, anti-inflammatory, and microcirculation-improving effects are still limited, and the specific molecular mechanism is still unclear. Since the pathogenesis of VUs mainly involves the extravasation of iron-containing hemoglobin and the increase in iron-containing erythrocyte numbers, for the first time, we used a hemin-induced HFF model of VUs to simulate the VU environment to the greatest extent, and we verified that hemin successfully induced ferroptosis via cell viability assays and by measuring FSP1, CoQ, GPX4, lipid peroxidation, Fe^2+^, GSH, and MDA levels. We found that DDL inhibited ferroptosis in HFFs by regulating the CoQ-FSP1 axis. We examined protein and mRNA expression in VU tissues that had been treated with DDL and found that ferroptosis was attenuated compared to the ferroptosis levels that were observed in untreated VU tissues. These findings provide a theoretical basis for understanding the molecular biological mechanism by which DDL functions in the treatment of VU.

Clinically, VUs were found to have symptoms of skin darkening and dark brown pigmentation, and we experimentally proved that the above symptoms of VUs were related to the increase in Fe^2+^ and that the increase in Fe^2+^ had a strong Fenton’s reaction to the formation of lipid peroxidation, which further aggravated the formation of ferroptosis. The symptoms of darkening of skin and dark brown pigmentation were reduced significantly after DDL treatment, and the Fe^2+^ was reduced compared with that of previous treatment. Therefore, we speculate that DDL may be a potential specific target or pathway for inhibiting ferroptosis caused by excess Fe^2+^. This specificity is associated with one particular manifestation of increased Fe^2+^ in VUs, which may need to be verified by many experiments at a later stage.

The ferroptosis inhibitory protein FSP1 was first identified by American and German scholars, and cellular overexpression of FSP1 significantly protects cells against the induction of ferroptosis by factors that trigger its initiation. Scholars have identified FSP1 inhibitor ferroptosis sensitizer 1 (FSEN1), which is a noncompetitive inhibitor that acts by selectively targeting and inhibiting FSP1 to sensitize cancer cells to ferroptosis (Elguindy and Nakamaru-Ogiso, 2015). In addition, synthetic lethality screens showed that FSEN1 functions synergistically with ferroptosis inducers containing endoperoxides, including dihydroartemisinin, to trigger ferroptosis. These results provide new tools for exploring the use of FSP1 as a therapeutic target and highlight the value of combination therapeutic regimens that target FSP1 and other ferroptosis defense pathways. Scholars found that the primary role of FSP1 in protecting A549 and H460 cells from ferroptosis was associated with high levels of FSP1-associated proteins, and it is noteworthy that FSEN1 induced a low level of ferroptosis in A375 melanoma cells in the absence of RSL3 combination therapy; these results suggest that A375 cells depend on FSP1 in a particularly strong manner to inhibit ferroptosis. The ability of FSEN1 to induce ferroptosis may depend on the expression levels of FSP1 and ferroptosis-related factors, such as GPX4, dihydroorotate dehydrogenase (DHODH), GTP cyclohydrolase 1 (GCH1), and ACSL4 (Hendricks et al., 2023).

The modification of FSP1 by myristoylation was identified due to the localization of FSP1 on lipid droplets as well as lipid membranes. Moreover, mutation of the cardamoylation modification site in FSP1 demonstrated that FSP1 can function in resistance to ferroptosis only when it has been modified by cardamoylation (Bersuker et al., 2019). FSP1 was found to serve as a biomarker of ferroptosis resistance in a variety of cancer cells. Experiments with H460 lung cancer cells revealed that the expression of FSP1 could maintain the growth of lung cancer cells when GPX4 was inactivated. Therefore, inhibitors of FSP1 may become drug candidates for cancer therapy.

Scholars (Doll et al., 2019) have explored the protein structural basis that allows FSP1 to inhibit ferroptosis. Based on previous work, investigators have found that the N-terminus of the FSP1 protein plays an important role in the inhibition of ferroptosis. The N-terminus of FSP1 contains a classical cardamylation modification-related motif that affects its interaction with the bilayer structure. The investigators mutated the N-terminus of FSP1 to construct a mutant FSP1 protein, namely, FSP1(G2A), and confirmed that the FSP1(G2A) protein does not undergo cardamomylation modification. The investigators further suggested that mutated FSP1 (G2A) did not inhibit ferroptosis. Furthermore, the inhibition of ferroptosis by FSP1 can be eliminated by pancoumaroyltransferase inhibitors. These results suggest that the FSP1 protein needs to be myristoylated to exert its inhibitory effect on ferroptosis (Doll et al., 2017).

Marcus ConradJose and other researchers found that FSP1 inhibits ferroptosis in a glutathione-independent manner (Tonnus et al., 2021). These researchers used an expression cloning approach to identify genes that could compensate for the absence of GPX4. These efforts identified FSP1 as a previously unknown ferroptosis resistance gene that confers unprecedented protection against ferroptosis caused by GPX4 deletion (Zhang et al., 2022).

Researchers further demonstrated that FSP1 can inhibit ferroptosis through CoQ10: its reduced form, namely, panthenol, traps lipid peroxyl radicals that mediate lipid peroxidation, and FSP1 catalyzes this process through regeneration using NAD(P)H. The pharmacological targeting of FSP1 can exert strong synergistic effects with GPX4 inhibitors, thereby triggering ferroptosis in many individuals with cancer. Immunoblotting analysis of FSP1-knockdown cells, such as FSP1-knockdown MDA436 cells, revealed that FSP1 knockdown decreases cellular resistance to RSL3-induced ferroptosis, while re-expression of FSP1 in mice restores cellular resistance to ferroptosis (Mao et al., 2021).

FSP1 and GPX4 constitute the two main ferroptosis defense systems; GPX4 is a phospholipid hydroperoxidase that uses GSH to detoxify lipid hydroperoxides and thus inhibit ferroptosis, whereas FSP1, which mainly localizes to the plasma membrane, acts as an oxidoreductase to reduce CoQ to CoQH2 (Mishima et al., 2022). CoQH2 then acts as a lipophilic radical-trapping antioxidant that detoxifies lipid peroxyl radicals. FSP1 promotes ferroptosis resistance in cancer cells by producing the antioxidant form of CoQ (Jin et al., 2023). Despite the important role of FSP1, there are few molecular tools that target the CoQ-FSP1 pathway (Koppula et al., 2022). CoQ and FSP1 were first identified in oncological diseases and used in antitumor therapy. We first showed that the CoQ-FSP1 pathway plays an important role in VU healing, and we found that DDL exerts important effects through this pathway. CoQ is a redox-active lipid that is synthesized in the inner mitochondrial membrane (IM) and limits lipid peroxidation and ferroptosis. CoQ10 has been shown to be a very good free radical-scavenging antioxidant that targets phospholipids and lipoproteins (Deshwal et al., 2023).

Scholars (Deshwal et al., 2023) have shown that the lipid transporter protein STARD7 is needed for CoQ synthesis in mitochondria and its transport to the cell membrane. In mitochondria, STARD7 maintains CoQ synthesis, oxidative phosphorylation and cristae morphogenesis, but in the cytosol, STARD7 is responsible for transporting CoQ to the cell membrane and preventing iron oxidation (Liang and Jiang, 2023). However, STARD7 overexpression in the cytosol increases cellular resistance to ferroptosis. Scientists at the Max Planck Institute for the Biology of Aging in Cologne, Germany, have identified a protein that is involved in transporting this antioxidant, which is mainly produced in mitochondria, to the cell membrane, where it protects against cell death by preventing free-radical-induced oxidative damage (Yang et al., 2014). CoQ is synthesized mainly in the inner mitochondrial membrane, where it acts as a carrier that transports electrons between respiratory chain complexes I and III or complexes II and III, is responsible for the transfer of electrons from complex I or II to complex III, and acts as a coenzyme for a variety of oxidoreductases that are involved in the *ab initio* synthesis of pyrimidine nucleotides, fatty acid β-oxidation or sulfide oxidation.

In addition, CoQ is a natural antioxidant that is involved in the neutralization of reactive oxygen radicals (ROS) that are generated by normal body metabolism. Despite the increasing importance of nonmitochondrial CoQ in cellular functions, it has been unclear how extremely hydrophobic CoQ molecules are transferred to the cytoplasmic membrane after being synthesized in the mitochondria (Zaki et al., 2018). This is indeed a problem that cells must face. After being formed in the mitochondria, antioxidants must reach the cell surface through the aqueous cytoplasm to neutralize the oxidized lipid material. In addition, the highly hydrophobic nature of CoQ means that our bodies absorb very little of this molecule from food. German scientists eventually discovered that a protein that is involved in the intracellular transport of phosphatidylcholine called STARD7 is also involved in the intracellular transfer of CoQ. This transport protein is located not only in the mitochondria but also in the cytoplasm. Through STARD7, mitochondria actively transport CoQ to the cell surface to protect against cell death. Again, this finding shows that mitochondria are not only important as suppliers of energy for our cells but also play a crucial regulatory role.

Anna Greka’s team elucidated the nonclassical, cell-specific manner by which CoQ functions independently of the electron transport chain (ETC). The study confirmed that CoQ deficiency caused by Pdss2 enzyme defects in podocytes leads to polyunsaturated fatty acid (PUFA) metabolism disorders and Braf/Mapk pathway disorders but not ETC., dysfunction. CoQ has important implications in the treatment of kidney diseases (Sidhom et al., 2021).

FSP1 and DHODH reduce CoQ to CoQH2 at the plasma membrane and inner mitochondrial membrane, respectively (Li et al., 2023); CoQH2 acts as a radical-trapping antioxidant that neutralizes lipid peroxyl radicals and thus inhibits ferroptosis. FIN56 induces ferroptosis not only through the degradation of GPX4 but also through interaction with squalene synthase (SQS) and depletion of CoQ. Statins are widely used to treat many diseases. Some statins have received FDA approval. Statins have been formulated as therapeutic nanoparticles that block 3-hydroxy-3-methylglutamine-CoA reductase (HMGCR) in the methanate pathway. When CoQ is reduced, ferroptosis subsequently occurs (Du and Guo, 2022).

We found that DDL significantly increased the cellular expression of FSP1 and CoQ, that the cellular mortality rate decreased after DDL treatment, that the lipid peroxidation level and Fe^2+^ level decreased after DDL treatment and that ferroptosis was attenuated after DDL treatment, as shown by flow cytometry. We first inhibited CoQ and found that this inhibition influenced the effect of DDL, and then we inhibited FSP1, which also influenced the effect of DDL. Then, we both overexpressed FSP1 and inhibited CoQ, which also influenced the effect of DDL.

GPX4, which is also known as phospholipid peroxylated glutathione peroxidase (PHGPx), is the fourth member of the selenium-containing GPX family (Xue et al., 2023). GPX4 has a molecular weight of approximately 19 kDa and consists of approximately 170 amino acids. Several GPX family members, including GPX1-GPX8, have been identified in mammals (Yao et al., 2023).

However, only GPX4 exhibited the ability to scavenge membrane lipid peroxide products, and this function is associated with the unique amino acid sequence and spatial structure of GPX4 (Friedmann Angeli et al., 2014). GPX4 was shown to be a key regulator of ferroptosis (Liu et al., 2022). GPX4 uses its catalytic activity to inhibit lipid peroxide toxicity and maintain homeostasis of the membrane lipid bilayer (Zou et al., 2019). Biochemically, ferroptosis is characterized by elevated iron ion levels, high ROS production, decreased GPX4 activity (Xu et al., 2021), and the accumulation of lipid metabolites. Numerous studies have shown that ferroptosis is closely associated with neoplastic diseases, neurodegenerative diseases, and organ damage (Xu et al., 2019). GPX4 levels in VU tissues were significantly lower than those in normal tissues, and GPX4 levels in VU tissues that were treated with DDL were significantly higher than those in untreated VU tissues; additionally, ferroptosis occurred in VU tissues, and this was attenuated by DDL treatment (Li et al., 2019).

ACSL4 is an important isoenzyme in the metabolism of polyunsaturated fatty acids (PUFAs), and increasing research on the mechanism underlying ferroptosis has identified ACSL4 as an important indicator of ferroptosis (Liao et al., 2022). Some scholars have found that ACSL4 promotes ferroptosis and is highly expressed in renal tissues in acute kidney injury (AKI) (Wang et al., 2022). ACSL4 promotes neuronal ferroptosis after OGD, and ACSL4 overexpression leads to reduced cell viability and increased lipid peroxidation (Cui et al., 2021). Ma Qiang (He et al., 2022)et al. found that ACSL4 plays an important role in exertional pyrexia, that ACSL4 plays a key role in promoting ferroptosis in skeletal muscle cells and that the downstream effector of the Hippo pathway, YAP/TEAD1/TEAD4, regulates ACSL4 transcription (Tuo et al., 2022), thus inducing ferroptosis in skeletal muscle. A significant increase in ACSL4 expression accompanied hemin-induced cellular ferroptosis; we then added DDL, and ACSL4 expression decreased.

However, the current model of VU in animals is far from perfect and is still in the exploratory stage; this led to a limitation in our study. We can only logically reach the conclusion that intervention can alter the VU healing process and reveal the important role of the corresponding molecules in VU healing. Further studies may require FSP1 transgenic mice to confirm the mechanism in detail; however, such animals are not currently available. Although DDL has received manufacturing approval and is in production, the drug composition is complex and requires individual studies for each metabolite, such as Dandelion Extract. In recent years, research on chronic wounds has focused on the use of various novel materials as wound dressings to promote chronic wound healing. DDL may be used as a metabolite in a novel wound dressing to promote wound healing; however, its physicochemical properties and solubility need to be improved.

## 5 Conclusion

In summary, this study found for the first time that high degrees of ferroptosis occur in VU tissues, and this effect is attenuated by DDL treatment. The CoQ-FSP1 pathway was shown to play an important role in the process of VU healing, and DDL could promote cell proliferation by activating CoQ and the downstream molecule FSP1, leading to an increase in CoQ and FSP1 levels while increasing GPX4 levels and attenuating cellular ferroptosis. These findings reveal the molecular biological mechanism by which DDL functions in the treatment of VU, promoting the pharmacological application of DDL, laying the foundation for its further clinical application, further elucidating the mechanism underlying VU healing and providing new ideas for approaches to promote wound repair.

## Data Availability

The original contributions presented in the study are included in the article/Supplementary material, further inquiries can be directed to the corresponding author.
